# Targeting FAPα-expressing hepatic stellate cells overcomes resistance to antiangiogenics in colorectal cancer liver metastasis models

**DOI:** 10.1172/JCI157399

**Published:** 2022-10-03

**Authors:** Ming Qi, Shuran Fan, Maohua Huang, Jinghua Pan, Yong Li, Qun Miao, Wenyu Lyu, Xiaobo Li, Lijuan Deng, Shenghui Qiu, Tongzheng Liu, Weiqing Deng, Xiaodong Chu, Chang Jiang, Wenzhuo He, Liangping Xia, Yunlong Yang, Jian Hong, Qi Qi, Wenqian Yin, Xiangning Liu, Changzheng Shi, Minfeng Chen, Wencai Ye, Dongmei Zhang

**Affiliations:** 1 College of Pharmacy, Jinan University, Guangzhou, China.; 2 The First Affiliated Hospital of Jinan University, Guangzhou, China.; 3 Guangdong Province Key Laboratory of Pharmacodynamic Constituents of TCM and New Drugs Research, and; 4 School of Traditional Chinese Medicine, Jinan University, Guangzhou, China.; 5 Sun Yat-sen University Cancer Center, Guangzhou, China.; 6 Department of Cellular and Genetic Medicine, School of Basic Medical Sciences, Fudan University, Shanghai, China.; 7 School of Medicine, Jinan University, Guangzhou, China.

**Keywords:** Gastroenterology, Therapeutics, Colorectal cancer, Drug therapy, Pericytes

## Abstract

Vessel co-option has been demonstrated to mediate colorectal cancer liver metastasis (CRCLM) resistance to antiangiogenic therapy. The current mechanisms underlying vessel co-option have mainly focused on “hijacker” tumor cells, whereas the function of the “hijackee” sinusoidal blood vessels has not been explored. Here, we found that the occurrence of vessel co-option in bevacizumab-resistant CRCLM xenografts was associated with increased expression of fibroblast activation protein α (FAPα) in the co-opted hepatic stellate cells (HSCs), which was dramatically attenuated in HSC-specific conditional *Fap*-knockout mice bearing CRCLM allografts. Mechanistically, bevacizumab treatment induced hypoxia to upregulate the expression of fibroblast growth factor–binding protein 1 (FGFBP1) in tumor cells. Gain- or loss-of-function experiments revealed that the bevacizumab-resistant tumor cell–derived FGFBP1 induced FAPα expression by enhancing the paracrine FGF2/FGFR1/ERK1/-2/EGR1 signaling pathway in HSCs. FAPα promoted CXCL5 secretion in HSCs, which activated CXCR2 to promote the epithelial-mesenchymal transition of tumor cells and the recruitment of myeloid-derived suppressor cells. These findings were further validated in tumor tissues derived from patients with CRCLM. Targeting FAPα^+^ HSCs effectively disrupted the co-opted sinusoidal blood vessels and overcame bevacizumab resistance. Our study highlights the role of FAPα^+^ HSCs in vessel co-option and provides an effective strategy to overcome the vessel co-option–mediated bevacizumab resistance.

## Graphical abstract

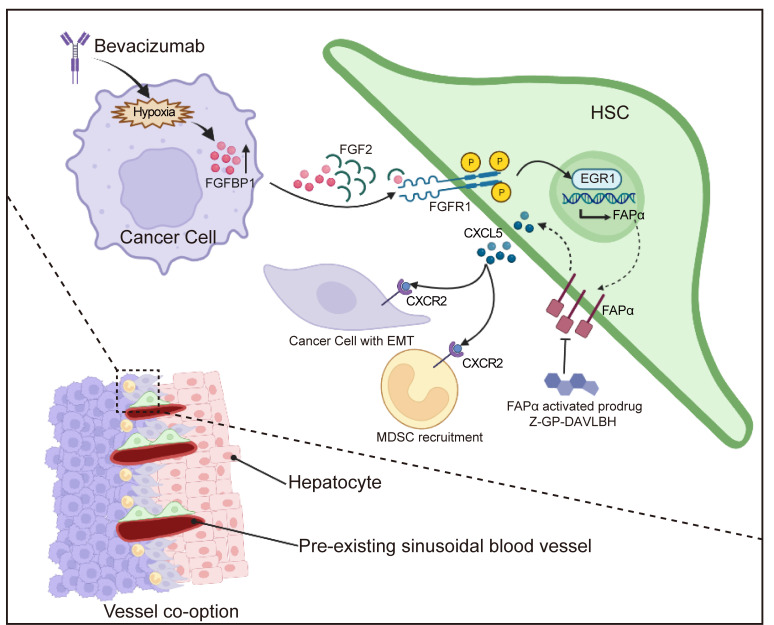

## Introduction

Colorectal cancer (CRC) is the third most common and the second most fatal malignancy in the world (1). Specifically, CRC liver metastasis (CRCLM) contributes to more than 70% of disease mortality (2). The angiogenesis inhibitor bevacizumab is commonly used in the treatment of CRCLM (3). However, intrinsic and acquired resistances frequently occur, resulting in treatment failure and cancer recurrence (4). CRCLM mainly contains 3 different histopathological growth patterns (HGPs): desmoplastic HGP (DHGP), pushing HGP (PHGP), and replacement HGP (RHGP) (5). These growth patterns have distinct histopathological features and utilize different mechanisms to obtain vascular supply. For DHGP and PHGP, angiogenesis is the main mode of blood supply for tumors. For RHGP, tumor cells infiltrate the hepatic plates of the liver parenchyma and hijack the preexisting sinusoidal vessels, which is referred to as vessel co-option (6). Vessel co-option has been considered an important mechanism mediating CRCLM resistance to antiangiogenic therapy (7). For example, bevacizumab or sorafenib combined with chemotherapeutic agents efficiently inhibits tumor neovascularization of CRCLM with DHGP and PHGP, but has a negligible effect on CRCLM with RHGP where vessel co-option occurs (8, 9). In addition, antiangiogenic therapy also increased the amount of co-opted blood vessels in several tumor xenograft models and clinical cancer cases (8–11). However, the molecular mechanisms underlying vessel co-option are poorly understood, and effective therapies for targeting vessel co-option are urgently needed. Currently, a few studies are primarily focused on the “hijacker” tumor cells in vessel co-option (12). For instance, overexpression of neuronal cell adhesion molecule L1 (L1CAM), serpin B1, actin-related protein 2/3 complex (ARP2/3), runt-related transcription factor-1 (RUNX1), and integrin α5β1 (ITGA5) in tumor cells facilitates vessel co-option by promoting the epithelial-mesenchymal transition (EMT) and motility of tumor cells (13–16). Nevertheless, the potential contribution of “hijackee” blood vessels to vessel co-option remains unknown.

Hepatic stellate cells (HSCs), also known as perisinusoidal cells, are a group of contractile and secretory cells closely attached to the periphery of hepatic sinusoid endothelial cells (17). HSCs are considered liver-specific pericytes and play a crucial role in the pathological process of CRCLM (17). CRC cells secrete multiple growth factors, including transforming growth factor β (TGF-β) to activate HSCs (18–21), which in turn secrete chemokines, cytokines, growth factors, or proteinases to enhance tumor growth, metastasis, angiogenesis, and immune escape in a positive feedback loop (17). Nevertheless, the potential roles of HSCs in vessel co-option remain unknown. Elucidating the molecular mechanism of vessel co-option from the perspective of HSCs will provide clues for novel targeted therapeutic strategies to overcome the vessel co-option–mediated resistance to antiangiogenic therapy.

Fibroblast activation protein α (FAPα) is a type II integral membrane serine protease that can specifically cleave N-terminal benzyloxy carbonyl–blocked (Z-blocked) Gly-Pro (Z-GP) dipeptide-linked substrates (22). FAPα is selectively expressed in tumor pericytes (23) or cancer-associated fibroblasts (CAFs) (24), which facilitates extracellular matrix remodeling, immunosuppression, and angiogenesis and subsequently promotes tumor progression and metastasis (25–27). The distinct dipeptide substrate hydrolytic activity of FAPα and its restricted expression in the tumor microenvironment make it an ideal target for an enzyme-activated prodrug strategy. Since HSCs are considered liver-specific pericytes, we here investigated the expression of FAPα in HSCs and its contribution to bevacizumab-induced vessel co-option. Our results showed that bevacizumab induced FAPα expression in HSCs through the fibroblast growth factor–binding protein 1 (FGFBP1)/FGF2/FGFR1/ERK1/-2/EGR1 axis and FAPα-promoted secretion of CXCL5 in HSCs, which activated CXCR2 to enhance myeloid-derived suppressor cell (MDSC) infiltration and tumor cell EMT to promote vessel co-option. Furthermore, targeting FAPα^+^ HSCs with the FAPα-activated prodrug Z-GP-DAVLBH (23) effectively overcame the vessel co-option–mediated CRCLM resistance to antiangiogenic therapy.

## Results

### FAPα is expressed in HSCs of co-opted sinusoidal blood vessels in bevacizumab-resistant CRCLM xenografts.

**Figure 1:**
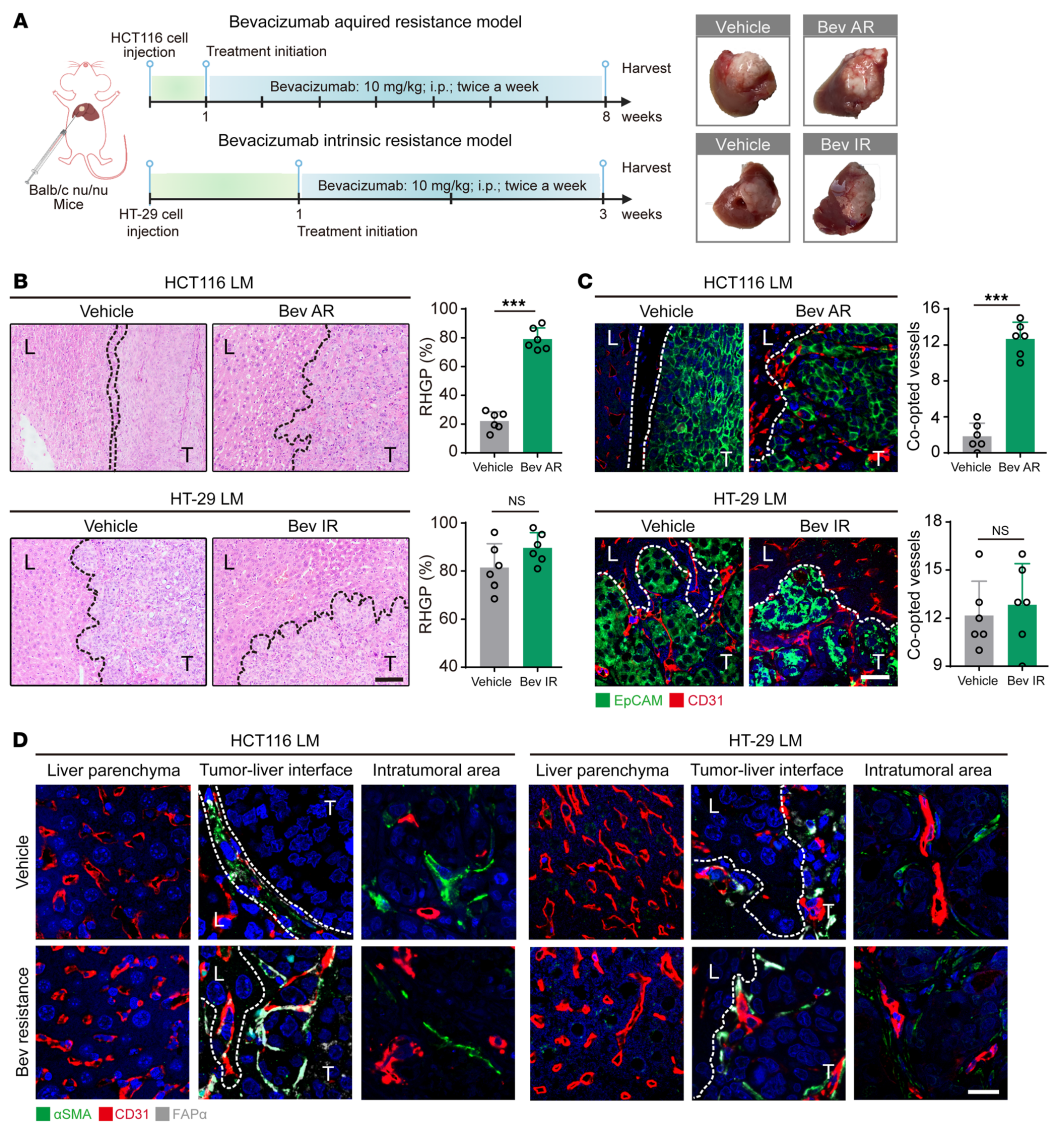
Bevacizumab treatment induces vessel co-option and FAPα expression in the co-opted HSCs in CRCLM xenografts. (**A**) Schematic showing the strategy for generating the bevacizumab-resistant CRCLM xenografts. Tumors were harvested and photographed at the end of experiments. (**B**) H&E staining of the tumor-liver interface of CRCLM xenografts. Scale bar: 100 μm. Quantification of RHGP is shown (*n* = 6). (**C**) Immunofluorescence staining of the EpCAM^+^ tumor cells (green) that infiltrated the liver parenchyma and hijacked the CD31^+^ sinusoidal blood vessels (red) in the tumor-liver interface of CRCLM xenografts. Scale bar: 50 μm. Quantification of the co-opted sinusoidal blood vessels is shown (*n* = 6). (**D**) Immunofluorescence staining of FAPα (gray) in αSMA^+^ HSCs (green) attached to the CD31^+^ sinusoidal blood vessels (red) in the CRCLM xenografts. Scale bar: 20 μm. Dotted lines indicate the tumor-liver interface. LM, liver metastases; T, tumor; L, liver; Bev AR, bevacizumab acquired resistance; Bev IR, bevacizumab intrinsic resistance. Data are presented as mean ± SEM. NS, no significance. ***P < 0.001 (2-tailed, unpaired *t* test).

The bevacizumab-resistant xenografts were first established to investigate the role of HSCs in vessel co-option. Bevacizumab-sensitive HCT116 CRCLM xenografts (28) were treated with bevacizumab (10 mg/kg) for 42 days to generate the acquired-bevacizumab-resistance models. The intrinsically bevacizumab–resistant HT-29 xenografts (29) were treated with bevacizumab (10 mg/kg) for 12 days to confirm the resistant profile (Figure 1A). The bevacizumab-resistant models were indicated by no significant differences in tumor growth ratio, Ki67 or PCNA proliferative index, and microvessel density (MVD) between vehicle- and bevacizumab-treated groups (Supplemental Figure 1, A–D; supplemental material available online with this article; https://doi.org/10.1172/JCI157399DS1). The HGPs in CRCLM xenografts were then examined, and our results showed that HGPs of HCT116 CRCLM xenografts in the vehicle group were mainly DHGP and PHGP, while RHGP was the main form in tumors from the bevacizumab-resistant group (Figure 1B). In contrast, HT-29 CRCLM xenografts in both vehicle- and bevacizumab-treated groups were mainly composed of the RHGP form (Figure 1B). The hijacking of sinusoidal blood vessels by tumor cells in RHGP lesions has been reported in both preclinical and clinical CRCLM cases (9, 11), and EpCAM^+^ tumor cells infiltrating the sinusoidal blood vessels are considered to indicate vessel co-option (8, 9, 11). Immunofluorescence results indicated that the number of co-opted sinusoidal blood vessels in acquired-bevacizumab-resistance HCT116 CRCLM xenografts was dramatically higher than that in the vehicle group (Figure 1C), while the number of co-opted sinusoidal blood vessels was equivalent in vehicle- and bevacizumab-treated HT-29 CRCLM xenografts (Figure 1C).

We further investigated changes in vascular characteristics in the co-opted sinusoidal blood vessels following bevacizumab resistance. Upregulation of FAPα in αSMA^+^ activated HSCs has been observed in liver fibrosis and cirrhosis (30, 31). We found that FAPα was expressed in αSMA^+^ HSCs on the co-opted sinusoidal blood vessels in the bevacizumab-resistant HCT116 CRCLM xenografts but not in the vehicle group (Figure 1D), whereas αSMA^+^ HSCs on the co-opted sinusoidal blood vessels in the vehicle- and bevacizumab-treated HT-29 CRCLM xenografts displayed strong FAPα staining. Conversely, the staining of FAPα and αSMA was absent in HSCs of normal liver tissue adjacent to carcinoma or in the angiogenic microvessels in the central area of CRCLM xenografts (Figure 1D). These data indicate that the vessel co-option–mediated intrinsic and acquired bevacizumab resistance in CRCLM might be associated with FAPα expression in HSCs in the co-opted sinusoidal blood vessels.

### FAPα induces CXCL5 secretion in HSCs to promote vessel co-option.

**Figure 2:**
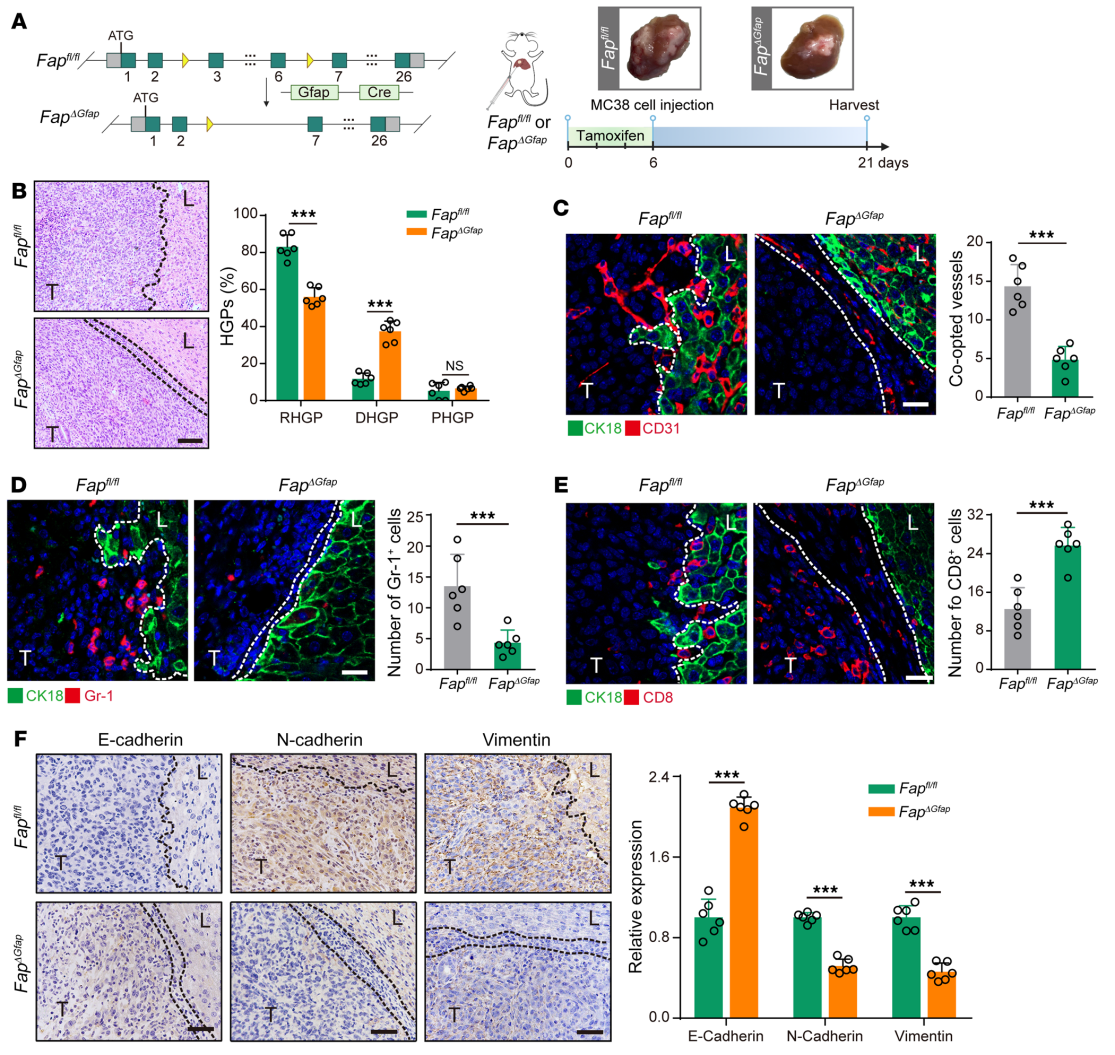
Conditional knockout of *Fap* in HSCs attenuates vessel co-option via inhibiting the recruitment of MDSCs, promoting the infiltration of CD8^+^ T cells, and suppressing tumor cell EMT. (**A**) Schematic showing the strategy for generating HSC-specific *Fap*-knockout mice (*Fap^ΔGfap^*), and the experimental design of tamoxifen-induced HSC-specific *Fap* knockout in MC38 CRCLM allografts established in *Fap^fl/fl^* or *Fap^ΔGfap^* mice. Tumors were harvested and photographed at the end of experiments. (**B**) H&E staining of the tumor-liver interface of MC38 CRCLM allografts. Scale bar: 100 μm. Quantification of HGPs is shown (*n* = 6). (**C**) Immunofluorescence staining of liver parenchyma (CK18^+^, green) and CD31^+^ sinusoidal blood vessels (red) in the tumor-liver interface of MC38 CRCLM allografts. Scale bar: 20 μm. Quantification of the co-opted sinusoidal blood vessels is shown (*n* = 6). (**D**) Immunofluorescence staining of liver parenchyma (CK18^+^, green) and Gr-1^+^ MDSCs (red) in the tumor-liver interface of MC38 CRCLM allograft tumors. Scale bar: 20 μm. Quantification of Gr-1^+^ MDSCs is shown (*n* = 6). (**E**) Immunofluorescence staining of liver parenchyma (CK18^+^, green) and CD8^+^ T cells (red) in the tumor-liver interface of MC38 CRCLM allograft tumors. Scale bar: 20 μm. Quantification of CD8^+^ T cells is shown (*n* = 6). (**F**) Immunohistochemical staining and quantification of E-cadherin, N-cadherin, and vimentin in the tumor-liver interface of MC38 CRCLM allografts (*n* = 6). Dotted lines indicate the tumor-liver interface. Scale bars: 50 μm. T, tumor. L, liver. Data are presented as mean ± SEM. NS, no significance. ***P < 0.001 (2-tailed, unpaired *t* test).

We next investigated the contribution of FAPα in HSCs to vessel co-option. Since MC38 allografts were intrinsically resistant to bevacizumab (Supplemental Figure 2A), this murine colon adenocarcinoma cell line was directly injected into the liver parenchyma of *Fap* wild-type mice (*Fap^fl/fl^*) or HSC-specific conditional *Fap*–knockout mice (*Fap^ΔGfap^*) to generate the intrinsically bevacizumab–resistant CRCLM allografts (Figure 2A and Supplemental Figure 2, B and C). Our results showed that MC38 CRCLM allografts in *Fap^fl/fl^* mice mainly consisted of RHGP and numerous co-opted sinusoidal blood vessels, which were significantly reduced in *Fap^ΔGfap^* mice (Figure 2, B and C). Tumor cell EMT and the establishment of an immune escape microenvironment are considered 2 important mechanisms mediating vessel co-option (32). Given that FAPα expression in CAFs can enhance the infiltration of MDSCs, impair antitumor T cell immunity (25), and promote tumor cell EMT (33), we speculated that FAPα^+^ HSCs may preestablish an immunosuppressive niche and induce tumor cell EMT to promote vessel co-option. As expected, the recruitment of Gr-1^+^ MDSCs was significantly suppressed, the frequency of CD8^+^ T cells was dramatically increased (Figure 2, D and E), and the expression of mesenchymal markers vimentin and N-cadherin was decreased alongside an increased expression of the epithelial marker E-cadherin (Figure 2F) in MC38 CRCLM allografts established in *Fap^ΔGfap^* mice compared with those in *Fap^fl/fl^* mice.

**Figure 3:**
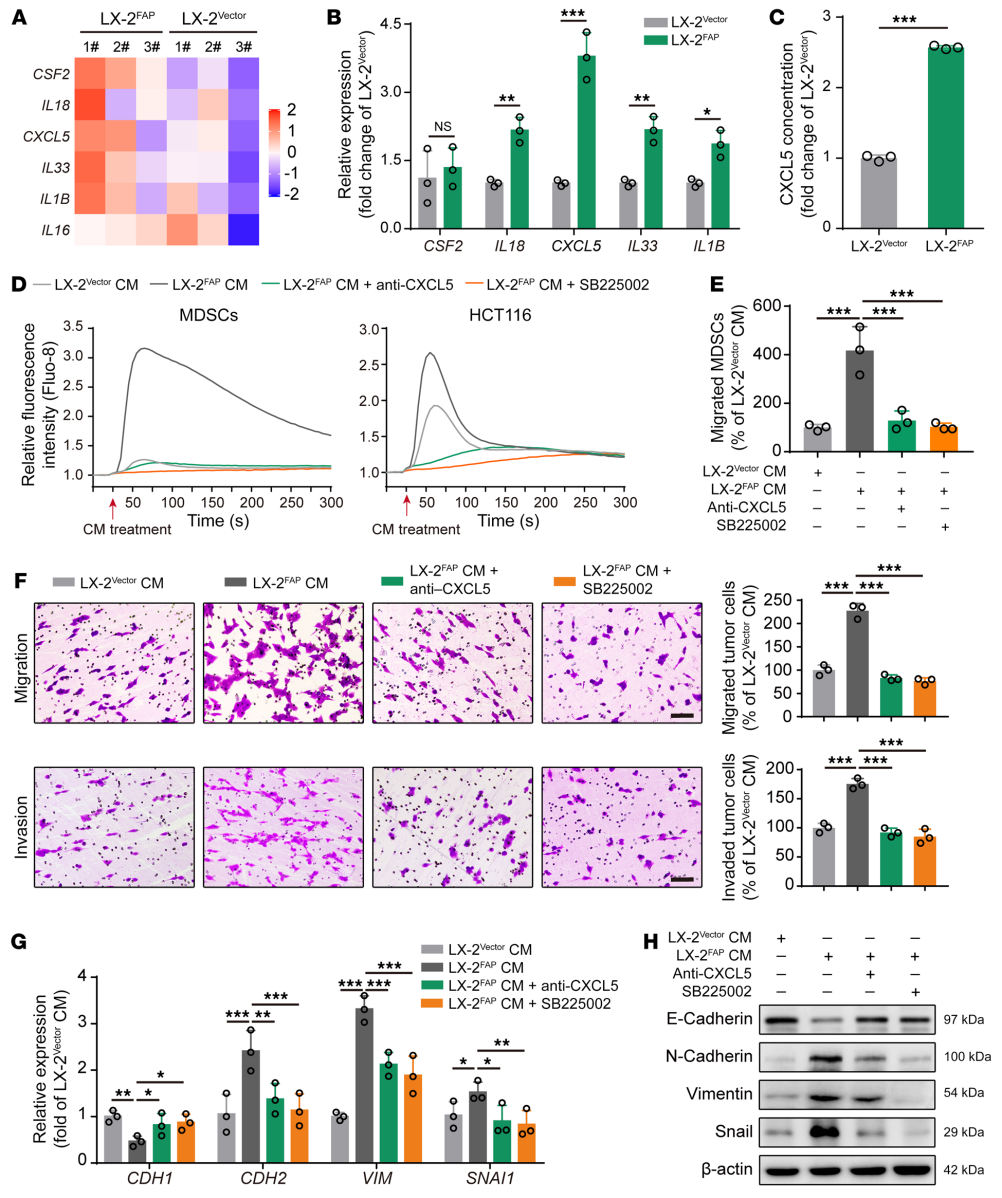
FAPα induces CXCL5 secretion in HSCs to promote MDSC recruitment and tumor cell EMT via activation of CXCR2. (**A**) Heatmap depicting the differentially expressed genes encoding secreted factors in LX-2 cells (fold change > 1.2, *P* < 0.05; *n* = 3). (**B**) RT-qPCR analysis of *CSF2*, *IL18*, *CXCL5*, *IL33*, and *IL1B* in LX-2 cells. (**C**) ELISA analysis of CXCL5 in LX-2 cells (*n* = 3). (**D**) Intracellular Ca^2+^ mobilization in MDSCs and HCT116 cells in response to the conditioned medium from LX-2 cells in the absence or presence of CXCL5-neutralizing antibody or SB225002. (**E**) Transwell assay for the migration of MDSCs treated with conditioned medium from LX-2 cells in the absence or presence of CXCL5-neutralizing antibody or SB225002 (*n* = 3). (**F**) Transwell assays for the migration and invasion of HCT116 cells treated with conditioned medium from LX-2 cells in the absence or presence of CXCL5-neutralizing antibody or SB225002 (*n* = 3). Scale bars: 100 μm. (**G**) RT-qPCR analysis of *CDH1*, *CDH2*, *VIM*, and *SNAI1* in HCT116 cells treated with conditioned medium from LX-2 cells in the absence or presence of CXCL5-neutralizing antibody or SB225002 (*n* = 3). (**H**) Western blotting analysis of E-cadherin, N-cadherin, vimentin, and snail in HCT116 cells treated with conditioned medium from LX-2 cells in the absence or presence of CXCL5-neutralizing antibody or SB225002. Data are presented as mean ± SEM. *P < 0.05; **P < 0.01; ***P < 0.001 (2-tailed, unpaired *t* test in **B** and **C**; 1-way ANOVA with Tukey’s post hoc comparison in **E**–**G**). CM, conditioned medium.

To further investigate the mechanisms underlying FAPα in HSC-regulated vessel co-option, the human HSC line LX-2 (34) with stable overexpression of FAPα (LX-2^FAP^) and the negative control cells (LX-2^Vector^) were generated (Supplemental Figure 3, A and B). RNA sequencing (RNA-seq) performed on LX-2^Vector^ and LX-2^FAP^ cells showed that the levels of 5 genes encoding secreted factors, including *CSF2*, *IL18*, *CXCL5*, *IL33*, *IL1B*, and *IL16* were significantly upregulated in LX-2^FAP^ cells (Figure 3A). A real-time quantitative polymerase chain reaction (RT-qPCR) assay confirmed that the levels of *IL18*, *CXCL5*, *IL33*, and *IL1B* were higher in LX-2^FAP^ cells than those in LX-2^Vector^ cells, and *CXCL5* was the most dramatically upregulated gene (Figure 3B). ELISA showed that the level of CXCL5 in the culture supernatant of LX-2^FAP^ cells was markedly higher than that in LX-2^Vector^ cells (Figure 3C), indicating that FAPα may be associated with the secretion of CXCL5 in HSCs. Given that tumor-derived CXCL5 promotes tumor cell EMT (35) and MDSC recruitment (36) by activating CXCR2, we proposed that HSC-derived FAPα promoted tumor cell EMT and MDSC recruitment through the CXCL5/CXCR2 axis. We found that the conditioned medium from LX-2^FAP^ cells stimulated a rapid and transient increase in intracellular Ca^2+^ mobilization in MDSCs and HCT116 cells compared with the conditioned medium from LX-2^Vector^ cells, which was significantly attenuated by a CXCL5-neutralizing antibody or SB225002 (a CXCR2 inhibitor) (Figure 3D and Supplemental Figure 3C). In addition, the conditioned medium from LX-2^FAP^ cells enhanced the migration of MDSCs, promoted the migration and invasion of HCT116 cells, increased the expression of vimentin, N-cadherin, and snail, and decreased the expression of E-cadherin in HCT116 cells (Figure 3, E–H). These effects were significantly attenuated by the CXCL5-neutralizing antibody or SB225002 (Figure 3, D–H). Taken together, our results show that FAPα induces CXCL5 secretion in HSCs to promote tumor cell EMT and MDSC recruitment through activation of CXCR2, thus facilitating vessel co-option.

### Tumor cell–derived FGFBP1 induces FAPα expression in HSCs to promote vessel co-option.

**Figure 4:**
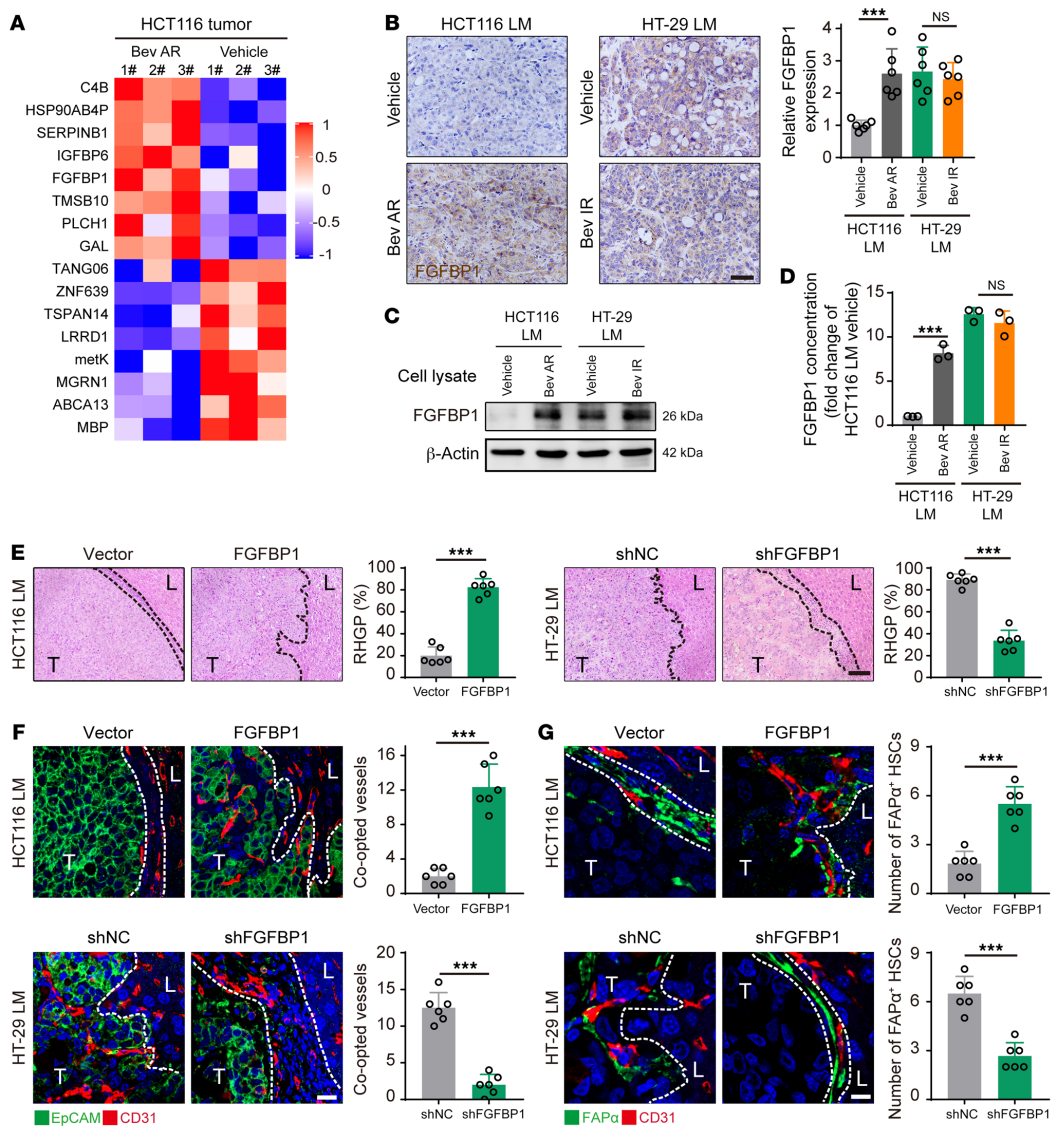
Tumor cell–derived FGFBP1 induces FAPα expression in HSCs to facilitate vessel co-option. (**A**) Differential proteomic analysis of tumor tissues obtained from bevacizumab-sensitive and -resistant HCT116 CRCLM xenografts (log_2_[fold change] > 1.0, *P* < 0.05; *n* = 3). (**B**) Immunohistochemical staining and quantification of FGFBP1 in the tumor-liver interface of CRCLM xenografts (*n* = 6). Scale bar: 50 μm. (**C** and **D**) Western blotting (**C**) and ELISA (**D**) analysis of the expression of FGFBP1 in HCT116 and HT-29 cells isolated from the indicated CRCLM xenografts (*n* = 3). (**E**) H&E staining of the tumor-liver interface of CRCLM xenografts. Scale bar: 100 μm. Quantification of RHGP is shown (*n* = 6). (**F**) Immunofluorescence staining of the EpCAM^+^ tumor cells (green) that infiltrated the liver parenchyma and hijacked the CD31^+^ sinusoidal blood vessels (red) in the tumor-liver interface of CRCLM xenografts. Scale bar: 20 μm. Quantification of the co-opted sinusoidal blood vessels is shown (*n* = 6). (**G**) Immunofluorescence staining of FAPα^+^ HSCs (green) attached to the CD31^+^ sinusoidal blood vessels (red) in the tumor-liver interface of CRCLM xenografts. Scale bar: 10 μm. Quantification of the co-opted FAPα^+^ HSCs is shown (*n* = 6). Dotted lines indicate the tumor-liver interface. LM, liver metastases; T, tumor; L, liver. Data are presented as mean ± SEM. NS, no significance. ***P < 0.001 (2-tailed, unpaired *t* test).

To investigate the mechanisms underlying the expression of FAPα in the co-opted HSCs, we analyzed the proteomic profiles of bevacizumab-sensitive and -resistant HCT116 CRCLM xenografts. According to the analysis of the UniProtKB/Swiss-Prot *Homo* *sapiens* proteome database, 17 upregulated proteins were identified in bevacizumab-resistant tumors (Figure 4A). RT-qPCR assay confirmed that the level of *FGFBP1* was the most notably upregulated gene in HCT116 cells derived from the bevacizumab-resistant CRCLM xenografts (Supplemental Figure 4, A and B). Additionally, immunohistochemical staining, Western blotting, and ELISA revealed that the expression of FGFBP1 in tumors or primary cultured tumor cells derived from bevacizumab-resistant HCT116 and HT-29 CRCLM xenografts was higher than in those derived from bevacizumab-sensitive HCT116 CRCLM xenografts (Figure 4, B–D).

To investigate whether tumor cell–derived FGFBP1 was essential for the expression of FAPα in HSCs and vessel co-option, HCT116 cells with low expression of FGFBP1 were transfected with lentivirus containing the vector or *FGFBP1* sequence to generate HCT116^Vector^ cells or FGFBP1-overexpressing HCT116^FGFBP1^ cells, respectively (Supplemental Figure 4, C and E). In contrast, HT-29 cells with high expression of FGFBP1 were transfected with lentivirus containing negative control shRNA (shNC) or *FGFBP1* shRNA (shFGFBP1) to generate HT-29^shNC^ cells or *FGFBP1*-knockdown HT-29^shFGFBP1^ cells, respectively (Supplemental Figure 4, D and E). Our results showed that HCT116^FGFBP1^ CRCLM xenografts had a higher ratio of RHGP compared with HCT116^Vector^ CRCLM xenografts, whereas HT-29^shFGFBP1^ CRCLM xenografts had a lower ratio of RHGP compared with HT-29^shNC^ CRCLM xenografts (Figure 4E and Supplemental Figure 5A). Consistently, the number of co-opted sinusoidal blood vessels in HCT116^FGFBP1^ and HT-29^shNC^ CRCLM xenografts was significantly higher than those in HCT116^Vector^ and HT-29^shFGFBP1^ CRCLM xenografts (Figure 4F). In addition, FAPα was highly expressed in HSCs in the co-opted sinusoidal blood vessels in HCT116^FGFBP1^ and HT-29^shNC^ CRCLM xenografts compared with those in HCT116^Vector^ and HT-29^shFGFBP1^ CRCLM xenografts, respectively (Figure 4G). Moreover, MDSC recruitment and tumor cell EMT in HCT116^FGFBP1^ and HT-29^shNC^ CRCLM xenografts were more prominent than those in HCT116^Vector^ and HT-29^shFGFBP1^ CRCLM xenografts (Supplemental Figure 5, B–D). Taken together, our results suggest that tumor cell–derived FGFBP1 induces FAPα expression in HSCs and promotes vessel co-option.

### Tumor cell–derived FGFBP1 induces FAPα expression in HSCs via the FGF2/FGFR1/ERK1/-2/EGR1 axis.

**Figure 5:**
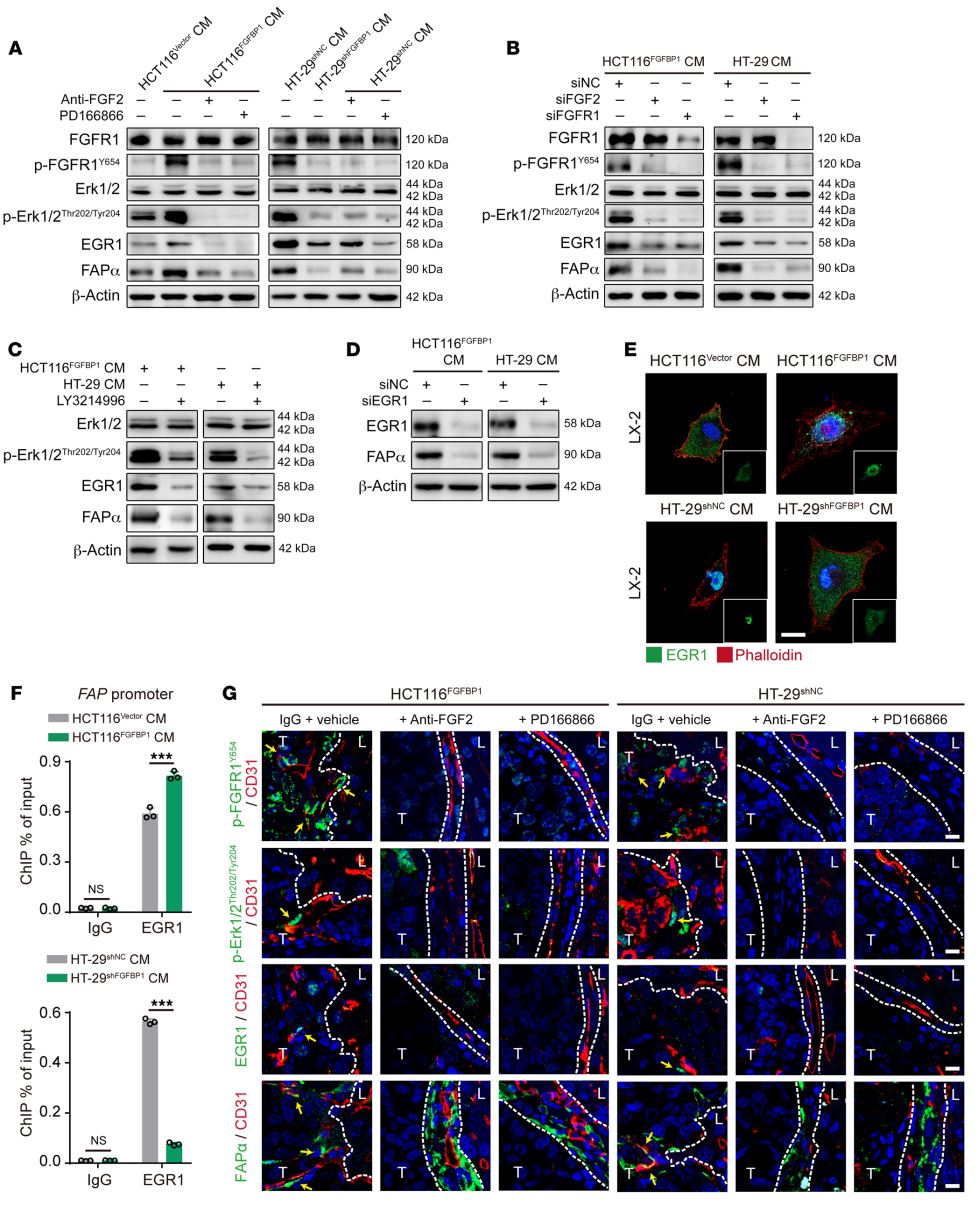
Tumor cell–derived FGFBP1 induces FAPα expression in HSCs via activating the FGF2/FGFR1/ERK1/-2/EGR1 axis. (**A**) Western blotting analysis of FGFR1, p-FGFR1, ERK1/-2, p-ERK1/-2, EGR1, and FAPα in LX-2 cells treated with the conditioned medium from HCT116 or HT-29 cells in the absence or presence of FGF2-neutralizing antibody or PD-166866. (**B**) Western blotting analysis of FGFR1, p-FGFR1, ERK1/-2, p-ERK1/-2, EGR1, and FAPα in the FGF2- or FGFR1-knockdown LX-2 cells treated with conditioned medium from HCT116 or HT-29 cells. (**C**) Western blotting analysis of ERK1/-2, p-ERK1/-2, EGR1, and FAPα in LX-2 cells treated with the conditioned medium from HCT116 or HT-29 cells in the absence or presence of LY3214996. (**D**) Western blotting analysis of EGR1 and FAPα in EGR1-knockdown LX-2 cells treated with the conditioned medium from HCT116 or HT-29 cells. (**E**) Immunofluorescence staining for the location of EGR1 in LX-2 cells treated with the conditioned medium from HCT116 or HT-29 cells. Scale bar: 20 μm. (**F**) ChIP-qPCR analysis of the binding of EGR1 to the *FAP* promoter in LX-2 cells treated with the conditioned medium from HCT116 or HT-29 cells (*n* = 3). (**G**) Immunofluorescence staining of p-FGFR1, p-ERK1/-2, EGR1, or FAPα in HSCs (green) attached to the CD31^+^ sinusoidal blood vessels (red) in the tumor-liver interface of CRCLM xenografts (*n* = 6). Scale bars: 10 μm. Yellow arrows indicate the p-FGFR1^+^, p-ERK1/-2^+^, EGR1^+^, or FAPα^+^ HSCs. Dotted lines indicate the tumor-liver interface. LM, liver metastases; CM, conditioned medium; T, tumor; L, liver. Data are presented as mean ± SEM. NS, no significance. ***P < 0.001 (1-way ANOVA with Tukey’s post hoc comparison).

FGFBP1 can enhance the activation of FGFR1 signaling by releasing FGF2 from the extracellular matrix (ECM) (37, 38). We found that the levels of *FGF2* and *FGFR1* in LX-2 cells were higher than other FGFs and FGFRs (Supplemental Figure 6, A and B). In addition, FGF2 and p-FGFR1 were highly expressed in FAPα^+^ HSCs in the co-opted sinusoidal blood vessels in bevacizumab-resistant HCT116 and HT-29 CRCLM xenografts (Supplemental Figure 6C), indicating that tumor cell–derived FGFBP1 might induce FAPα expression in HSCs by activating the FGF2/FGFR1 signaling pathway. We next investigated the underlying mechanism by which the tumor cell–derived FGFBP1-triggered activation of the FGF2/FGFR1 axis induced FAPα expression in HSCs. Given that the ERK1/-2/EGR1 axis has been demonstrated to be downstream of FGFR1 (39), and EGR1 has been shown to induce FAPα expression by binding to the *FAP* promoter (40), we proposed that the FGFR1-activation-induced FAPα expression in HSCs might be regulated by the ERK1/-2/EGR1 axis. Our results showed that the phosphorylation of FGFR1 and ERK1/-2 and the expression of EGR1 and FAPα were significantly increased in LX-2 cells treated with the conditioned medium from HCT116^FGFBP1^ and HT-29^shNC^ cells compared with those in LX-2 cells treated with the conditioned medium from HCT116^Vector^ and HT-29^shFGFBP1^ cells (Figure 5A). The above effects were significantly attenuated by an FGF2-neutralizing antibody, PD-166866 (an FGFR1-specific inhibitor), or knockdown of *FGF2* and *FGFR1* in LX-2 cells (Figure 5, A and B, and Supplemental Figure 7, A and B). In addition, LY3214996 (an ERK1/-2–specific inhibitor) treatment significantly abrogated the expression of EGR1 and FAPα in LX-2 cells treated with conditioned medium from HCT116^FGFBP1^ or HT-29 cells (Figure 5C). Moreover, knockdown of *EGR1* significantly decreased the expression of FAPα in LX-2 cells that were treated with the conditioned medium from HCT116^FGFBP1^ or HT-29 cells (Figure 5D and Supplemental Figure 7C). Furthermore, the translocation of EGR1 into the nucleus in LX-2 cells treated with the conditioned medium from HCT116^FGFBP1^ or HT-29^shNC^ cells was higher than that in LX-2 cells treated with the conditioned medium from HCT116^Vector^ or HT-29^shFGFBP1^ cells (Figure 5E). ChIP-qPCR assay showed that the binding of EGR1 to the *FAP* promoter was significantly upregulated in LX-2 cells treated with conditioned medium from HCT116^FGFBP1^ or HT-29^shNC^ cells compared with that in LX-2 cells treated with conditioned medium from HCT116^Vector^ or HT-29^shFGFBP1^ cells (Figure 5F).

The in vivo experiments also demonstrated that the FGF2-neutralizing antibody or PD-166866 significantly inhibited the expression of p-FGFR1, p-ERK1/-2, EGR1, and FAPα in HSCs in HCT116^FGFBP1^ or HT-29^shNC^ CRCLM xenografts (Figure 5G and Supplemental Figure 8A). As a result, FGF2-neutralizing antibody or PD-166866 treatment significantly decreased the ratio of RHGP lesions (Supplemental Figure 8B) and the number of co-opted sinusoidal blood vessels (Supplemental Figure 8C). These data indicate that tumor cell–derived FGFBP1 induces FAPα expression in HSCs through the FGF2/FGFR1/ERK1/-2/EGR1 axis.

### FGF2/FGFR1/FAPα/CXCL5 axis in HSCs is responsible for the bevacizumab-induced vessel co-option.

**Figure 6:**
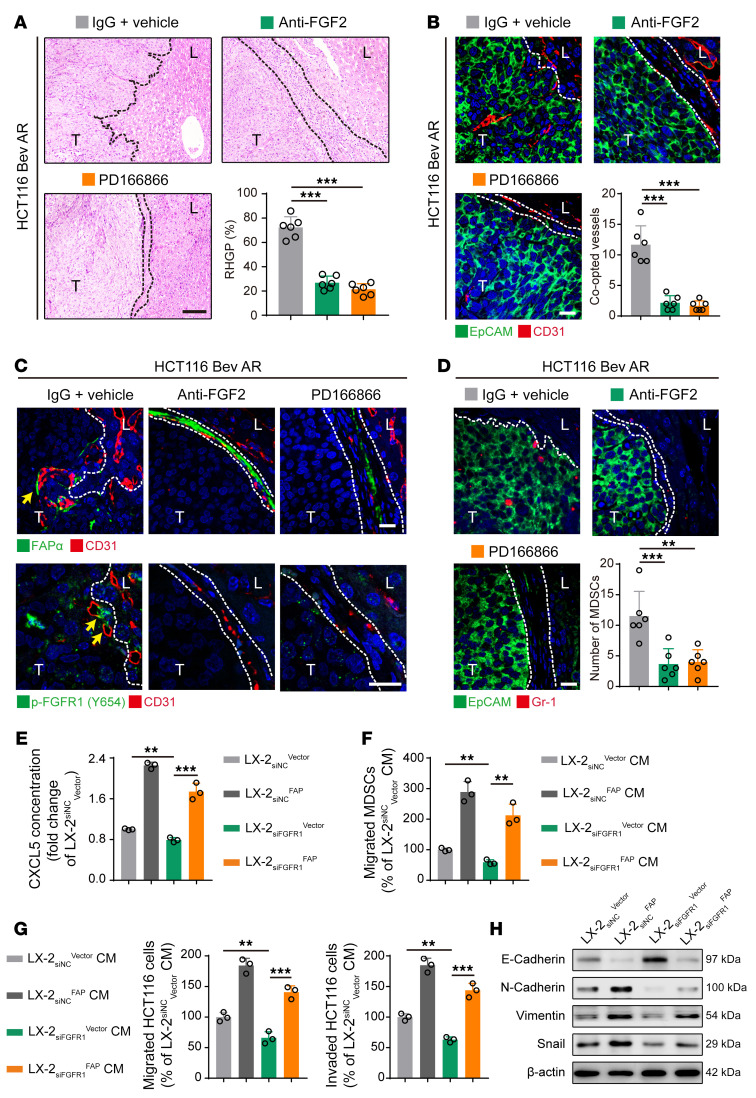
FGF2/FGFR1/FAPα axis is essential for the secretion of CXCL5 in HSCs, tumor cell EMT, and MDSC recruitment. (**A**) H&E staining of the tumor-liver interface of HCT116 CRCLM xenografts. Scale bar: 100 μm. Quantification of RHGP is shown (*n* = 6). (**B**) Immunofluorescence staining of the EpCAM^+^ tumor cells (green) that infiltrated the liver parenchyma and hijacked the CD31^+^ sinusoidal blood vessels (red) in the tumor-liver interface of HCT116 CRCLM xenografts. Scale bar: 20 μm. Quantification of the co-opted sinusoidal blood vessels is shown (*n* = 6). (**C**) Immunofluorescence staining of FAPα^+^ (green) or p-FGFR1^+^ (green) HSCs attached to the CD31^+^ sinusoidal blood vessels (red) in the tumor-liver interface of HCT116 CRCLM xenografts. Scale bars: 20 μm. (**D**) Immunofluorescence staining of the EpCAM^+^ (green) tumor cells and Gr-1^+^ MDSCs (red) in the tumor-liver interface of HCT116 CRCLM xenografts. Scale bar: 20 μm. Quantification of the Gr-1^+^ MDSCs is shown (*n* = 6). (**E**) ELISA analysis of CXCL5 concentration in the culture medium of LX-2 cells. (**F**) Transwell assay for the migration of MDSCs treated with the conditioned medium from LX-2 cells (*n* = 3). (**G**) Transwell assays for the migration and invasion of HCT116 cells treated with the conditioned medium from LX-2 cells (*n* = 3). (**H**) Western blotting analysis of E-cadherin, N-cadherin, vimentin, and snail in HCT116 cells treated with the conditioned medium from LX-2 cells. Dotted lines indicate the tumor-liver interface. Bev AR, bevacizumab acquired resistance; CM, conditioned medium; T, tumor; L, liver. Data are presented as mean ± SEM. **P < 0.01; ***P < 0.001 (1-way ANOVA with Tukey’s post hoc comparison).

We further investigated the role of the FGF2/FGFR1/FAPα pathway in promoting bevacizumab-induced vessel co-option. Our results showed that the combination of bevacizumab with either the FGF2-neutralizing antibody or PD-166866 significantly inhibited tumor growth and reduced the MVD in the bevacizumab-resistant HCT116 and HT-29 CRCLM xenografts (Supplemental Figure 9, A–C). In addition, FGF2-neutralizing antibody or PD-166866 markedly decreased the ratio of RHGP (Figure 6A and Supplemental Figure 10A) and the number of co-opted sinusoidal blood vessels (Figure 6B and Supplemental Figure 10B). Both the FGF2-neutralizing antibody and PD-166866 inhibited the expression levels of p-FGFR1 and FAPα in HSCs (Figure 6C and Supplemental Figure 10C), suppressed the recruitment of Gr-1^+^ MDSCs (Figure 6D and Supplemental Figure 10D), decreased the expression of vimentin, and increased the expression of E-cadherin in tumor cells (Supplemental Figure 10E). These data indicated that the activation of the FGF2/FGFR1/FAPα axis might be responsible for bevacizumab-induced vessel co-option by promoting tumor cell EMT and MDSC recruitment.

To investigate the underlying mechanisms of the activation of the FGF2/FGFR1/FAPα axis in promoting tumor cell EMT and MDSC recruitment, the *FGFR1*-knockdown LX-2 cells were transfected with vector (LX-2_siNC_^Vector^, LX-2_siFGFR1_^Vector^) or FAPα-overexpressing plasmid (LX-2_siFGFR1_^Vector^, LX-2_siFGFR1_^FAP^) (Supplemental Figure 11A). ELISA showed that knockdown of *FGFR1* significantly decreased the expression of CXCL5, as indicated by the significantly lower level of CXCL5 in the culture medium of LX-2 _siFGFR1_^Vector^ cells than in the culture medium of LX-2_siNC_^Vector^ cells (Figure 6E). As a result, compared with the culture medium from LX-2_siNC_^Vector^, the LX-2_siFGFR1_^Vector^ cell culture medium suppressed the migration of MDSCs and the migration and invasion of HCT116 cells (Figure 6, F and G, and Supplemental Figure 11B), decreased the expression of vimentin, N-cadherin, and snail, and increased the expression of E-cadherin in HCT116 cells (Figure 6H and Supplemental Figure 11C). However, these inhibitory effects were fully rescued by the overexpression of FAPα, evidenced by the levels of CXCL5 in the culture medium of LX-2_siFGFR1_^FAP^ cells that were significantly higher than those in the culture medium of LX-2_siFGFR1_^Vector^ cells (Figure 6E). Moreover, the LX-2_siFGFR1_^FAP^ cell culture medium promoted the migration of MDSCs and the migration and invasion of HCT116 cells (Figure 6, F and G, and Supplemental Figure 11B), increased the expression of vimentin, N-cadherin, and snail, and decreased the expression of E-cadherin in HCT116 cells compared with the medium from LX-2_siFGFR1_^Vector^ (Figure 6H and Supplemental Figure 11C). These findings indicate that FGFR1 activation may be dependent on FAPα to induce secretion of CXCL5 in HSCs and to promote tumor cell EMT and MDSC recruitment, thus facilitating vessel co-option.

### Bevacizumab-induced FAPα expression in HSCs is associated with vessel co-option in patients with CRCLM.

**Figure 7:**
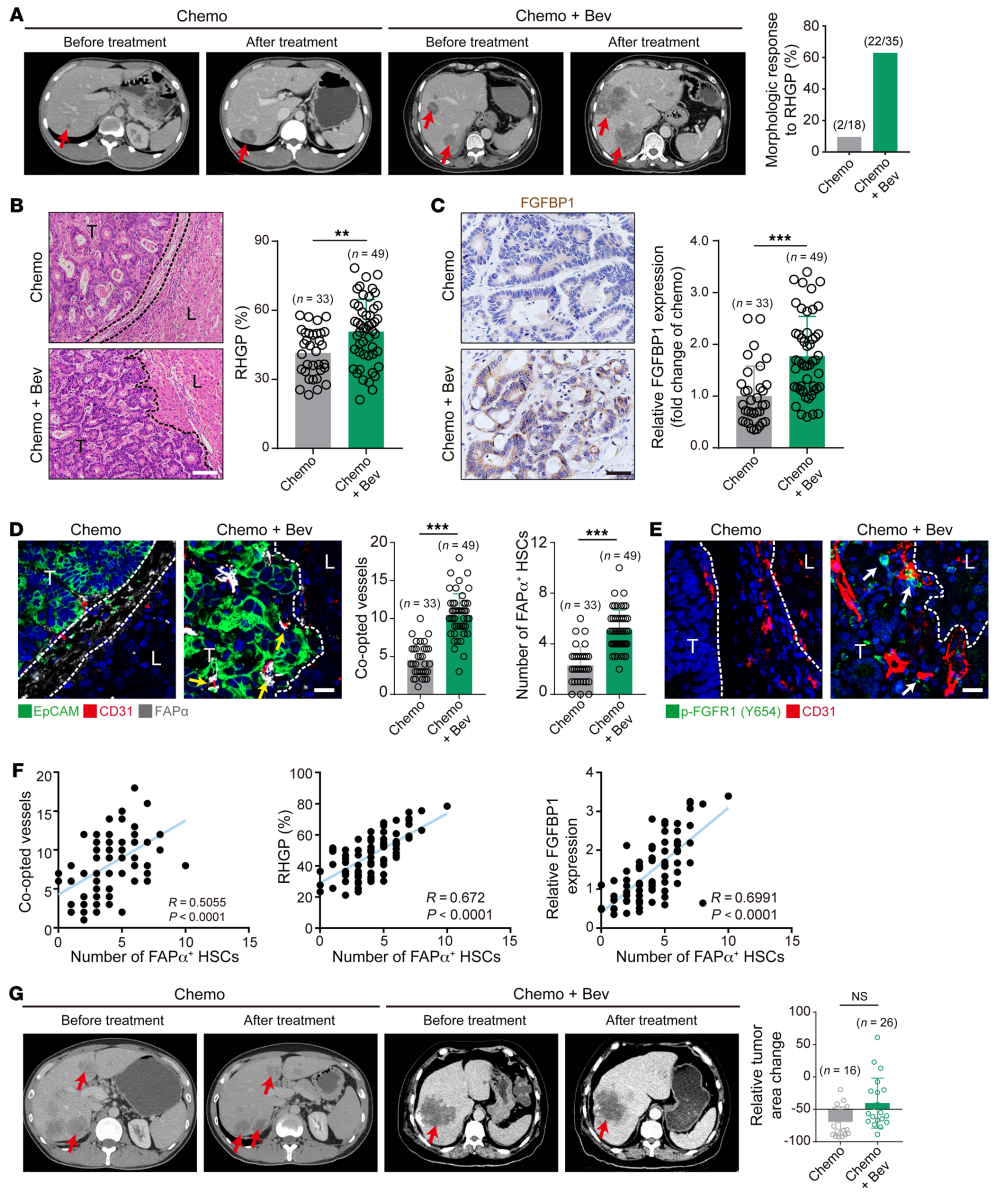
Bevacizumab induces vessel co-option to mediate treatment resistance in CRCLM patients. (**A**) CT scan images of CRCLM patients with DHGP or PHGP treated preoperatively with Chemo or Bev+Chemo, and red arrows indicate the tumor lesions. Quantification of the morphologic response to RHGP is shown. (**B**) H&E staining of the tumor-liver interface of human CRCLM tissues. Quantification of RHGP is shown (*n* = 6). Scale bar: 100 μm. (**C**) Immunohistochemical staining of FGFBP1 in the tumor-liver interface of human CRCLM tissues. Scale bar: 20 μm. Quantification of FGFBP1 staining is shown (right panel, *n* = 6). (**D**) Immunofluorescence staining of the EpCAM^+^ cancer cells (green) that infiltrated the liver parenchyma and hijacked the CD31^+^ sinusoidal blood vessels (red), and FAPα (gray) expression in the co-opted HSCs in the tumor-liver interface of human CRCLM tissues. Scale bar: 20 μm. Quantification of the co-opted sinusoidal blood vessels and FAPα^+^ HSCs is shown (*n* = 6). Yellow arrows indicate the FAPα^+^ HSCs. (**E**) Immunofluorescence staining of p-FGFR1(green) in HSCs attached to the CD31^+^ sinusoidal blood vessels (red) in the tumor-liver interface of human CRCLM tissues. Scale bar: 20 μm. White arrows indicate the p-FGFR1^+^ HSCs. (**F**) Pearson’s correlation analysis of FAPα^+^ HSCs and the co-opted sinusoidal blood vessels, percentage of RHGP, or FGFBP1 expression in human CRCLM tissues (*n* = 82). (**G**) CT scan images of CRCLM patients with RHGP treated preoperatively with Chemo or Bev+Chemo, and red arrows indicate the tumor lesions. Quantification of the change of tumor burden is shown. Dotted lines indicate the tumor-liver interface. Chemo, chemotherapy; Bev, bevacizumab; T, tumor; L, liver. Data are presented as mean ± SEM. NS, no significance. **P < 0.01, ***P < 0.001 (2-tailed, unpaired *t* test).

The above results demonstrated that FAPα in HSCs was associated with vessel co-option in animal models. However, the correlation of FAPα in HSCs with bevacizumab-induced vessel co-option in patients with CRCLM remains unknown. As shown in CT scans of patients with CRCLM (Figure 7A), the morphology of lesions in patients with DHGP or PHGP treated preoperatively with chemotherapy combined with bevacizumab (Chemo+Bev group) was significantly transformed into RHGP, but not with chemotherapy alone (Chemo group). Consistently, histopathological examination of tumor tissues derived from patients with CRCLM revealed that DHGP and PHGP were mainly present in the Chemo group, whereas patients treated with Chemo+Bev primarily contained RHGP (Figure 7B). Tumors in patients with Chemo+Bev treatment displayed stronger expression of FGFBP1 than those in patients treated with Chemo (Figure 7C). Chemo+Bev treatment resulted in increases in co-opted sinusoidal blood vessels and the number of FAPα^+^ HSCs compared with Chemo treatment (Figure 7D). HSCs in the co-opted sinusoidal blood vessels of patients treated with Chemo+Bev displayed a higher level of p-FGFR1 than in those treated with Chemo (Figure 7E). Additionally, the number of FAPα^+^ HSCs was correlated positively with FGFBP1 expression, co-opted sinusoidal blood vessels, and RHGP in CRCLM patients (Figure 7F). Furthermore, CRCLM patients with RHGP poorly responded to bevacizumab, as evidenced by the tumor burden of patients treated with Chemo+Bev that was increased similarly to those treated with Chemo (Figure 7G). Taken together, these data demonstrate that bevacizumab treatment resulted in the activation of the FGFBP1/FGFR1/FAPα axis, which may be responsible for vessel co-option–mediated bevacizumab resistance.

### Targeting FAPα^+^ HSCs disrupts co-opted sinusoidal blood vessels to overcome bevacizumab resistance.

**Figure 8:**
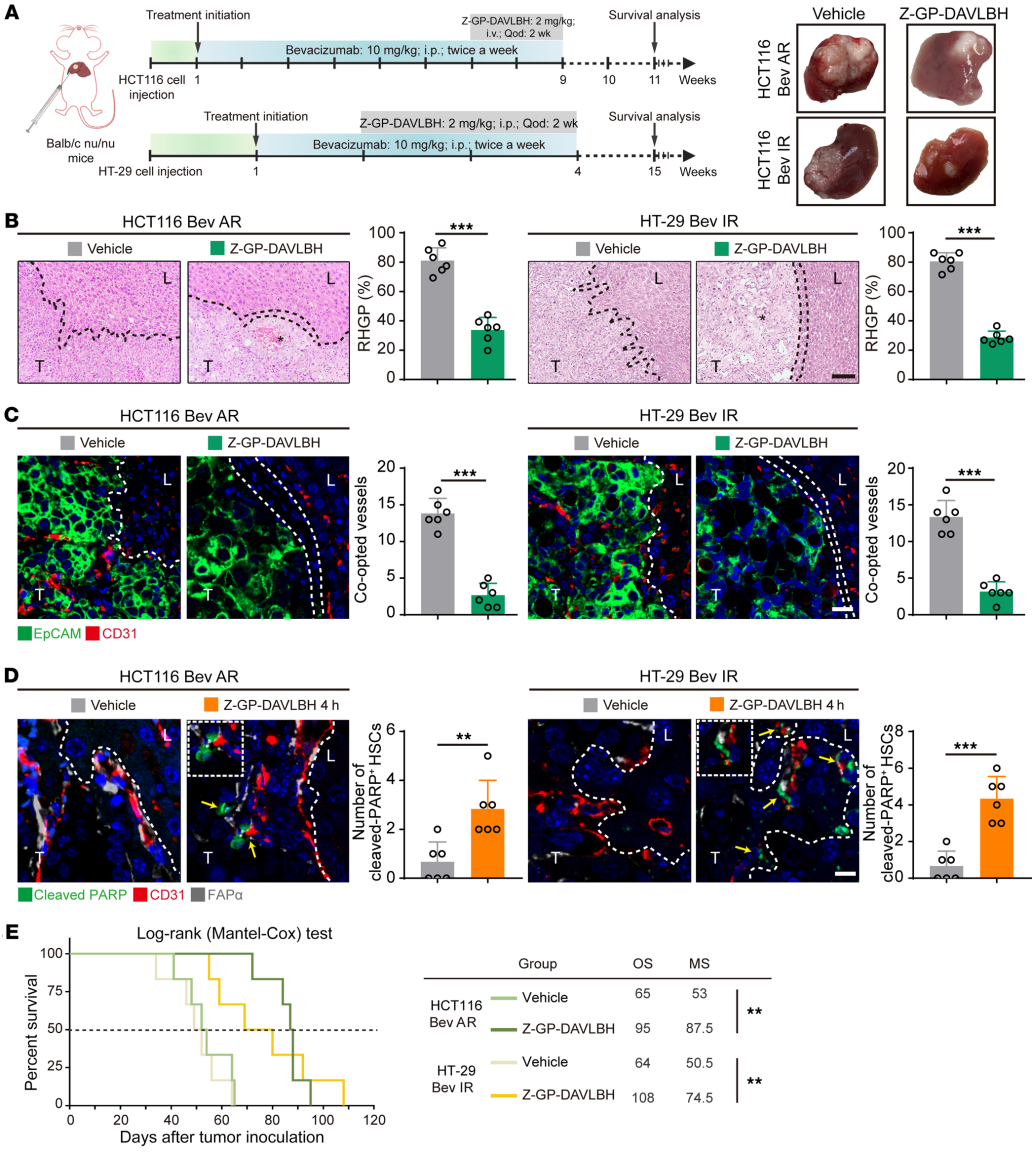
Targeting FAPα^+^ HSCs disrupts the co-opted sinusoidal blood vessels to overcome bevacizumab treatment resistance. (**A**) Therapeutic schedules for bevacizumab (10 mg/kg) and Z-GP-DAVLBH (2 mg/kg) treatment in mice bearing CRCLM xenografts. Tumors were harvested and photographed at the end of experiments. (**B**) H&E staining of the tumor-liver interface of CRCLM xenografts after Z-GP-DAVLBH treatments. Quantification of RHGP is shown (*n* = 6). Scale bar: 100 μm. Asterisks indicate tumor necrosis. (**C**) Immunofluorescence staining of the EpCAM^+^ tumor cells (green) that infiltrated the liver parenchyma and hijacked the CD31^+^ sinusoidal blood vessels (red) in the tumor-liver interface of CRCLM xenografts. Scale bar: 20 μm. Quantification of the co-opted sinusoidal blood vessels is shown (*n* = 6). (**D**) Immunofluorescence staining of the cleaved-PARP^+^ (green) apoptotic FAPα^+^ HSCs (gray) attached to CD31^+^ sinusoidal blood vessels (red) in the tumor-liver interface of CRCLM xenografts. Scale bar: 10 μm. Yellow arrows indicate the apoptotic cells. Quantification of cleaved-PARP^+^ HSCs is shown (*n* = 6). (**E**) The overall survival curves of mice bearing bevacizumab-resistant CRCLM xenografts treated with vehicle and Z-GP-DAVLBH (*n* = 6). OS, overall survival; MS, median survival; Bev AR, bevacizumab acquired resistance; Bev IR, bevacizumab intrinsic resistance. Data are presented as mean ± SEM. **P < 0.01; ***P < 0.001 (2-tailed, unpaired *t* test).

Next, we investigated whether targeting FAPα^+^ HSCs in the co-opted sinusoidal blood vessels by utilizing Z-GP-DAVLBH, an FAPα-activated prodrug synthesized by our lab (23), can eliminate the bevacizumab-induced vessel co-option. Our results showed that Z-GP-DAVLBH treatment induced tumor regression in both the bevacizumab-resistant HCT116 and HT-29 CRCLM xenografts, as indicated by the increased necrotic areas (Figure 8, A and B) and the decrease in MVD in tumor tissues (Supplemental Figure 12A). Mechanistically, Z-GP-DAVLBH treatment reversed the development of RHGP (Figure 8, A and B), decreased the amount of co-opted sinusoidal blood vessels (Figure 8C), blocked the recruitment of Gr-1^+^ MDSCs, and suppressed tumor cell EMT as indicated by the increase in E-cadherin and decrease in vimentin (Supplemental Figure 12, B–D). In addition, Z-GP-DAVLBH treatment disrupted the co-opted sinusoidal blood vessels and resulted in hemorrhaging in the liver-tumor interface (Supplemental Figure 12E). Such effects may be associated with the Z-GP-DAVLBH–induced apoptosis in FAPα^+^ HSCs of co-opted sinusoidal blood vessels (Figure 8D). Consistently, Z-GP-DAVLBH (1 μM) treatment dramatically inhibited proliferation and induced apoptosis in FAPα^+^ LX-2 cells (Supplemental Figure 13, A and B). Ultimately, Z-GP-DAVLBH prolonged the overall survival of the mice bearing intrinsically bevacizumab–resistant HT-29 xenografts (from 64 days to 108 days) and acquired-bevacizumab-resistance HCT116 CRCLM xenografts (from 65 days to 95 days) (Figure 8E). These data indicate that Z-GP-DAVLBH selectively induces apoptosis in FAPα^+^ HSCs, disrupts the co-opted sinusoidal blood vessels, and overcomes vessel co-option–mediated bevacizumab treatment resistance.

## Discussion

Vessel co-option, as a non–angiogenesis-dependent phenomenon, is commonly detected in liver cancer, lung cancer, renal cancer, and glioblastoma (41, 42). Accumulating evidence demonstrates that vessel co-option plays an important role in mediating resistance to antiangiogenic therapy (7). Currently, studies of vessel co-option are prevalently focused on the hijacker tumor cells (12), while effective strategies to overcome the treatment resistance are still lacking. Strikingly, the role of the hijackee sinusoidal blood vessels in vessel co-option remains largely unknown. Here, we elucidated the underlying mechanism of vessel co-option by the hijackee and uncovered an effective strategy to overcome the vessel co-option–mediated CRCLM resistance to antiangiogenic therapy. Bevacizumab treatment induced FAPα expression in HSCs and promoted tumor cell EMT and MDSC recruitment, which in turn significantly facilitated vessel co-option. Blockade of the FGFBP1/FGF2/FGFR1 signaling pathway inhibited FAPα expression in HSCs and attenuated vessel co-option, and targeting FAPα^+^ HSCs with the FAPα-activated prodrug Z-GP-DAVLBH can disrupt the co-opted sinusoidal blood vessels, thus eliminating vessel co-option.

Vessel co-option is a complex process involving multiple cell types, such as tumor cells, endothelial cells, pericytes, and immune cells. The key regulatory roles of tumor cells in vessel co-option have also been widely demonstrated by previous studies (13–16). Tumor cells hijacking blood vessels can induce expression of angiopoietin 2 and VEGF in vascular endothelial cells, which subsequently results in regression of the blood vessels (43). However, the effects of tumor cells on pericytes in co-opted vessels remain largely unknown. Recently, single-cell RNA-seq analysis of lung metastases of breast cancer revealed that sunitinib treatment can lead to a reduction in angiogenic pericytes and an increase in quiescent pericytes accompanied by the occurrence of vessel co-option (44). Conversely, our study revealed that bevacizumab treatment induced activation of HSCs and subsequently favored vessel co-option, which may be associated with the fact that HSCs are more susceptible to the fibrogenic response to liver injury or inflammatory stimuli (45). Furthermore, for cancer cells to travel alongside and hijack the preexisting blood vessels, they must preestablish an immunosuppressive microenvironment to ensure cell survival (32). It has been revealed that tumor-associated macrophages infiltrate when vessel co-option occurs (44). Here, we elucidated the mechanism and role of activated HSCs in the formation of the immune escape microenvironment through promoting MDSC recruitment and inhibiting CD8^+^ T cell infiltration. Taken together, this study enriches the perspective on the mechanism underlying vessel co-option.

The intercellular crosstalk between liver sinusoidal endothelial cells (LSECs) and HSCs has been demonstrated to exert critical regulation in maintaining the liver’s physiological function in liver fibrosis and cirrhosis (46). Tumor-induced activation of LSECs contributes to tumor immune evasion, angiogenesis, and metastasis (46). Tumor cells secrete TGF-β to activate HSCs, which in turn promote tumor progression by collagen deposition, promotion of tumor cell motility, and angiogenesis (47). However, the interaction between LSECs and HSCs in tumor progression is undefined. Given that the target of bevacizumab is tumor vascular endothelial cells, the sustaining bevacizumab treatment may cause genomic changes in LSECs in the co-opted sinusoidal blood vessels, which may act on both tumor cells and HSCs to promote vessel co-option. RNA-seq indicated that the genome-wide gene expression was markedly different between bevacizumab-resistant and -sensitive LSECs. However, coculture with LSECs had negligible effects on the expression of FGFBP1 in tumor cells and the tumor cell–primed expression of FAPα in HSCs (Supplemental Figure 14). Nevertheless, the above data were obtained from preliminary in vitro experiments, and the interaction between LSECs and HSCs in vivo is still unclear. Whether LSECs play an accomplice role in vessel co-option still needs further research.

Autocrine FGF2 and the high expression of FGFR1 in HSCs (48) and the activation of the FGF2/FGFR1 signaling pathway in HSCs have been demonstrated to play an important role in the regulation of liver fibrosis, cirrhosis, and tumor progression. For instance, FGF2 activates FGFR1 signaling in HSCs and upregulates the level of the *CyGB* gene to inhibit the transformation of HSCs into myofibroblasts and thereby alleviates liver fibrosis and cirrhosis (49). Low-molecular-weight FGF2 attenuates hepatic fibrosis by epigenetic downregulation of delta-like 1 (50), which is critical for hepatic fibrosis (51). However, genetic depletion of FGF2 (52) or pharmacological blockade of the FGFR1 pathway (53, 54) in HSCs significantly inhibits carbon tetrachloride– or lipopolysaccharide-induced hepatic cirrhosis and fibrosis. In addition, tumor cell–derived FGF2 activates a profibrotic phenotype in HSCs, which stimulates ECM synthesis (55). These paradoxical observations suggest that FGF2/FGFR1 signaling may play different regulatory roles in HSCs under different pathological conditions. Nevertheless, the role of the FGF2/FGFR1 signaling pathway in vessel co-option is still unknown. FGFBP1 is a chaperone protein that releases ECM-bound FGFs and promotes their binding to FGFRs, thereby enhancing the activation of the FGF/FGFR signaling pathway (37, 38). Although FGFBP1 expression in tumor cells is critical for the development of pancreatic, colon, and liver cancers (56–58), the molecular mechanisms by which FGFBP1 promotes the activation of HSCs remain unclear. Here, we demonstrated that bevacizumab treatment induced tumor hypoxia to upregulate the expression of FGFBP1 in tumor cells (Supplemental Figure 15), which activated the FGF2/FGFR1 signaling pathway to promote HSCs’ transformation into multiple phenotypes, including an “immunogenic” phenotype (Supplemental Figure 16) as reported previously (59). These findings indicate that tumor cell–derived FGFBP1–induced FAPα expression in HSCs could be an important regulatory mechanism for vessel co-option, and they also provide a potential therapeutic target to overcome CRCLM resistance to antiangiogenic therapy. However, as FGFR1 is also expressed on tumor cells, we here cannot exclude the contribution of the activation of FGFBP1/FGF2/FGFR1 signaling in tumor cells for vessel co-option.

Although vessel co-option has been known for more than 20 years (41), effective strategies to inhibit or disrupt vessel co-option are still lacking. Alternatively, genetic depletion of L1CAM, serpin B1, ARP2/-3, RUNX1, or ITGA5 in tumor cells has been demonstrated to attenuate vessel co-option (13–16). Angiogenesis inhibitors are ineffective in mature vessels with high pericyte coverage, which is mainly due to the prosurvival effect of pericytes on endothelial cells (60). The co-opted vessels are vasculatures with an intact structure and high pericyte coverage, which may be an important reason for vessel co-option–mediated angiogenesis inhibitor resistance. As HSCs are known as liver-specific pericytes (17, 61), the FAPα^+^ HSCs in co-opted vessels could be an attractive target for vessel co-option. Our data indicate that vessel co-option–mediated resistance to antiangiogenic therapy can be effectively abrogated by the FAPα-activated prodrug Z-GP-DAVLBH, which sheds light on the strategy for overcoming vessel co-option.

In conclusion, our findings reveal the molecular mechanism of HSCs in vessel co-option, in which HSCs mediate CRCLM resistance to bevacizumab treatment. In addition, this study provides a potential therapeutic strategy and drug candidate for overcoming vessel co-option–mediated bevacizumab resistance.

## Methods

### Human CRC liver metastasis tissue specimens.

A total of 81 liver metastases from patients with CRCLM were obtained from the First Affiliated Hospital of Jinan University. Detailed patient information is summarized in Supplemental Tables 1–3.

### Cell lines and cell culture.

Human CRC cell lines (HCT116 and HT-29) were obtained from ATCC. The HSC line LX-2 was purchased from Sure Biological Technology, and the mouse CRC cell line (MC38) was purchased from BeNa Culture Collection. These cell lines were cultured in DMEM (Gibco) with 10% FBS (ExCell Bio) and 1% penicillin-streptomycin (Gibco) at 37°C in a humidified atmosphere containing 5% CO_2_. All cell lines were authenticated as having no cross contamination of other human cell lines using the STR Multi-Amplification Kit (Microreader 21 ID System). All cell lines were tested negative for mycoplasma using the Mycoplasma Detection Set (M&C Gene Technology). For hypoxia experiments, cells at 60% confluence were transferred into a sealed hypoxia chamber filled with 5% CO_2_, 1% O_2_ and 94% N_2_ at 37°C and cultured for the indicated times.

### Animals.

The HSC-specific conditional *Fap*–knockout mice were generated as described previously with some modifications (62). Male BALB/c-Nu mice (aged 4–6 weeks), C57BL/6-*Fap^fl/fl^* mice (T052266), and Gfap-Cre mice that were backcrossed with C57BL/6J mice (T004857) were obtained from GemPharmatech. *Fap^ΔGfap^* (Gfap-*Fap* knockout) mice were generated by crossing Gfap-Cre mice and *Fap^fl/fl^* mice, and littermate *Fap^fl/fl^* mice were used as wild-type controls. All mice were maintained in a specific pathogen–free facility. The genotypes of transgenic mice were identified by PCR analysis of genomic DNA from tail snips using specific primers (Supplemental Table 4).

### Cell transfection.

Lentivirus containing luciferase vector GV260 (Ubi-MCS-firefly Luciferase-IRES-Puromycin), lentivirus containing either a FAPα or FGFBP1 overexpression plasmid and the corresponding vector GV367 (Ubi-MCS-SV40-EGFP-IRES-puromycin), and lentivirus containing shFGFBP1 plasmid and the corresponding vector GV248 (hU6-MCS-Ubiquitin-EGFP-IRES-puromycin) were constructed by Genechem. Lentivirus infection was carried out according to the manufacturer’s instructions. HCT116-luc cells were generated by infection with lentivirus containing luciferase vector. LX-2 cells stably overexpressing FAPα were obtained by infection with lentivirus containing FAPα overexpression plasmid. HCT116 cells stably overexpressing FGFBP1 were obtained by infection with lentivirus containing FGFBP1 overexpression plasmid. *FGFBP1*-knockdown HT-29 cells were generated by infection with *FGFBP1* shRNA lentivirus. Single-character and stable gene overexpression or knockdown cell clones were selected with 2 μg/mL puromycin (Life Technologies). HCT116 cells were transfected with pCMV3-*HIF1A* and empty plasmid using Lipofectamine 3000 (Thermo Fisher Scientific) according to the manufacturer’s instructions. For FGF2-, FGFR1-, EGR1-, and HIF1α-knockdown experiments, LX-2 cells were transfected with siRNA targeting *FGF2*, *FGFR1*, *EGR1*, or negative control, and HCT116 cells were transfected with siRNA targeting *HIF1A* or negative control (Genepharma). The sequences of shFGFBP1 are listed in Supplemental Table 5, and the sequences of siFGF2, siFGFR1, siEGR1, siHIF1A are listed in Supplemental Table 6. The transfection efficiency was determined by RT-qPCR and Western blotting assays.

### Construction of CRCLM xenograft tumors.

The HT-29 (11) or HCT116 (63) CRCLM xenografts were established as described previously with minor modifications. In brief, HT-29, HCT116, or HCT116-luc cells were resuspended in Matrigel at a concentration of 1 × 10^7^ cells/mL, and a total of 2 × 10^5^ tumor cells in 20 μL were injected into the left main lobe of the mouse liver. Tumor-bearing mice were randomized into the vehicle and bevacizumab groups on the seventh day. The treatment group of HCT116 or HCT116-luc xenografts was injected intraperitoneally (i.p.) with 10 mg/kg bevacizumab (Roche) twice a week for 7 weeks, and HT-29 xenografts were treated with 10 mg/kg bevacizumab for 2 weeks, while the vehicle group received IgG. In HCT116 or HCT116-luc CRCLM xenografts, tumors were collected on the 14th, 28th, and 42nd day after bevacizumab treatment to determine the characteristics of acquired bevacizumab resistance. To assess tissue hypoxia status, 50 mg/kg hypoxyprobe-1 (Hypoxyprobe, Inc.) was intravenously (i.v.) injected into mice for 30 minutes before being euthanized by CO_2_ asphyxiation. For therapeutic administration, mice bearing acquired-bevacizumab-resistance HCT116 xenografts and intrinsically bevacizumab–resistant HT-29 xenografts were treated with bevacizumab combined with i.v. injection of 2 mg/kg Z-GP-DAVLBH once every other day for 14 days, or combined with 1.8 mg/kg FGF2-neutralizing antibodies (Sigma-Aldrich) once a week for 2 weeks (i.v.), or combined with 20 mg/kg PD166866 (Selleck) once every other day for 3 weeks (i.p.). MC38 cells (2 × 10^5^ cells/mouse) were injected into the liver of *Fap^ΔGfap^* and *Fap^fl/fl^* mice after administering tamoxifen (100 mg/kg) via oral gavage once every other day for a total of 3 times, and the tumor-bearing mice were sacrificed by CO_2_ asphyxiation 2 weeks later. HCT116^Vector^, HCT116^FGFBP1^, HT-29^shNC^, and HT-29^shFGFBP1^ cells were injected into the left main liver lobe of male BALB/c nude mice. On the seventh day, HCT116^FGFBP1^ and HT-29^shNC^ mice were treated with 20 mg/kg PD166866 once every other day for 3 weeks (i.p.) or 1.8 mg/kg FGF2-neutralizing antibodies once a week for 2 weeks (i.v.). Tumor tissues were collected for further histological analysis.

### Bioluminescence imaging of tumor growth.

Bioluminescence imaging was conducted to monitor tumor growth in vivo using the IVIS Lumina LT imaging system (PerkinElmer). Mice bearing HCT116-luc CRCLM xenografts were injected (i.p.) with 150 mg/kg D-luciferin (Yeasen) before isoflurane (RWD Life Science) anesthesia. Live animal imaging was acquired 10 minutes after the injection of D-luciferin. For the quantification of total radiance efficiency, a region of interest was drawn around the tumor and radiance efficiency was measured.

### Histology, immunohistochemistry, and immunofluorescence.

Formalin-fixed tissue samples of mouse or human CRC liver metastasis tissues were embedded in paraffin and sectioned at a thickness of 5 μm. Hematoxylin and eosin (H&E) staining was performed according to standard procedures. The types of histopathological growth patterns were evaluated using ImageJ software (NIH), and the full-length pixels of RHGP (*a*), DHGP (*b*), and PHGP (*c*) in the liver-tumor interface of each image were calculated and converted into micrometers. The percentage RHGP was then quantified according to the following formula: *a*/(*a* + *b* + *c*) × 100. The same calculation method was used for the quantification of DHGP and PHGP. For immunohistochemical staining, deparaffinization, rehydration, antigen retrieval, permeabilization, and blocking were performed on the sections, followed by incubation of primary antibodies overnight at 4°C. The slides were then incubated with HRP-conjugated secondary antibodies and visualized using a DAB staining kit (Servicebio). Immunofluorescence staining was visualized with iF488-Tyramide, iF555-Tyramide, or iF647-Tyramide using TSAPLus Fluorescence Kits (Servicebio). Immunofluorescence staining of LX-2 cells was performed using Alexa Fluor 488–conjugated secondary antibodies (Invitrogen) and Alexa Fluor 594–conjugated phalloidin (Invitrogen). Image analyses were performed using an Olympus BX53 inverted epifluorescence microscope or a Zeiss LSM 800 confocal microscope. All positive cells were counted in high-power fields at a magnification of ×200, ×400, or ×640. Five areas from each section were randomly selected to count the percentage of positively stained cells and to calculate the mean staining extent using Image-Pro Plus 6.0 software (Media Cybernetics). Detailed information on the primary and secondary antibodies is listed in Supplemental Table 7.

### Isolation and characterization of tumor cells, LSECs, or MDSCs.

Human or mouse CRC liver metastatic tissues were harvested aseptically from CRCLM patients or tumor-bearing mice and then digested with a tumor dissociation kit (Miltenyi Biotec). A single-cell suspension of tissues was obtained using a GentleMAC tissue processor (Miltenyi Biotec) according to the manufacturer’s instructions. Tumor cells were isolated using anti-EpCAM beads (Miltenyi Biotec) and characterized as EpCAM^+^ (Biolegend) with purity greater than 95% by flow cytometry. LSECs were isolated using anti-CD146 beads (Miltenyi Biotec) and characterized as CD146^+^ (Miltenyi Biotec) with purity greater than 95% by flow cytometry as described previously (64). MDSCs were characterized as negative for HLA-DR (Biolegend) and Lin (Biolegend) but positive for CD33 (Biolegend) and CD11b (Biolegend), which were sorted by flow cytometry as described previously (65).

### Transcriptome sequencing and data analysis.

Total RNA was extracted using TRIzol Reagent (Servicebio) at a ratio of 10^6^ cells/mL of reagent according to the manufacturer’s instructions. High-throughput full transcriptome sequencing and subsequent bioinformatics analysis were performed by Applied Protein Technology Co., Ltd. In brief, mRNA was purified from total RNA using a NEBNext Poly(A) mRNA Magnetic Isolation Module (New England Biolabs, E7490) according to the manufacturer’s instructions. RNA-seq libraries were generated using ribosomal RNA–depleted RNAs with the NEBNext Ultra II RNA Library Prep Kit (New England Biolabs) following the manufacturer’s instructions. Libraries were controlled for quality and quantified using the Bioanalyzer 2100 system (Agilent Technologies). RNA-seq Illumina Libraries were prepared and sequenced on an Illumina GAIIx sequencer with 101 base-length read chemistry. For the data analysis, raw data in FASTQ format were processed through in-house Perl scripts. All downstream analyses were based on high-quality clean data. The index of the reference genome was built using Hisat2 v2.0.5 (http://daehwankimlab.github.io/hisat2/), and the paired-end clean reads were aligned to the reference genome using Hisat2 v2.0.5. For the quantification of gene expression level, FeatureCounts v1.5.0-p3 (https://sourceforge.net/projects/subread/files/subread-2.0.0/) was used to count the read numbers mapped to each gene. The fragments per kilobase of transcript per million mapped reads values of each gene were calculated based on the length of the gene and read count mapped to this gene. Analysis of differential expression was performed using the DESeq2 R package v1.16.1 (https://bioconductor.org/packages/release/bioc/html/DESeq2.html), and the differentially expressed mRNAs and genes were selected with statistical significance (*P* < 0.05) by R package edgeR. The volcano plot revealed the distributions of log_2_(fold change) and *P* values for the differentially expressed genes. The GO terms (http://www.geneontology.org) of these differentially expressed genes were annotated.

### Tandem mass tag quantitative proteomic analysis.

Tandem mass tag (TMT) quantitative proteomic analysis was performed as described previously with some modifications (66). Bevacizumab-sensitive and –acquired resistant HCT116 xenograft tumor samples (*n* = 3 per group) were homogenized in SDT buffer (4% SDS, 100 mM Tris-HCl, pH 7.6, 0.1 M DTT) and quantified using the BCA protein assay reagent (Thermo Fisher Scientific). Then, 20 μg of protein per sample was resolved by SDS-PAGE and digested according to the FASP (filter-aided sample preparation) procedure (67). After that, digested peptides were labeled according to the instructions of the TMT labeling kit (Thermo Fisher Scientific), and the TMT-labeled peptide of each sample was mixed in equal amounts and fractionated using high-pH reversed-phase liquid chromatography. For proteomic analysis, the fractionated peptides were loaded onto the loading column (Thermo Fisher Scientific Acclaim PepMap 100, 100 μm × 2 cm, nanoViper C18) in 95% buffer A (0.1% v/v formic acid in water) and separated on a nanoflow HPLC Easy-nLC system (Thermo Fisher Scientific, 10 cm, ID 75 μm, 3 μm, C18-A2) at a flow rate of 300 nL/min (buffer A, 0.1% v/v formic acid in water; buffer B, 0.1% (v/v) formic acid in acetonitrile). The elution program was optimized as follows: 3% buffer B, 0–5 minutes; 3%–28% buffer B, 5–95 minutes; 28%–38% buffer B, 95–110 minutes; 38%–100% buffer B, 110–115 minutes; 100% buffer B, 115–120 minutes. Samples were analyzed using a Q-Exactive mass spectrometer (Thermo Fisher Scientific). The mass spectrometry data were analyzed using Mascot version 2.2 (http://www.matrixscience.com) and Proteome Discoverer version 1.4 (http://www.thermoscientific.com/en/product/proteome-discoverer-software.html) and searched against the UniProtKB/Swiss-Prot *Homo* *sapiens* proteome database (www.uniprot.org; downloaded on January 5, 2018, including 56,695 sequences). For protein quantitative analysis, the relevant parameters and descriptions were as follows: enzyme = trypsin, max missed cleavages = 2, fixed modification = carbamidomethyl (C), variable modifications = oxidation (M), peptide false discovery rate (FDR) ≤ 0.01. The protein ratios were calculated based on the median of only unique peptides of the protein. Proteins with fold change greater than 1.2 or less than 0.83 and *P* value (Student’s *t* test) less than 0.05 were considered differentially expressed.

### RT-qPCR assay.

Total RNAs were extracted using the E.Z.N.A. Total RNA Kit I (Omega Bio-Tek) and reverse transcribed to cDNA using the All-in-One cDNA Synthesis SuperMix (Bimake) according to the manufacturers’ instructions. RT-qPCR was performed using SYBR Green I Master (Genestar) according to the manufacturer’s instructions. The relative mRNA expression level of target genes was normalized to the endogenous *ACTB* gene using the 2^–ΔΔCt^ method. All primers are shown in Supplemental Table 8.

### ChIP-qPCR assay.

ChIP assays were conducted using the SimpleChIP Enzymatic Chromatin IP Kit according to the manufacturer’s instruction (Cell Signaling Technology). Antibodies are listed in Supplemental Table 9. The primers used for the RT-qPCR analysis of precipitated DNA are listed in Supplemental Table 10. The signal relative to input was calculated using a standard formula as follows: percentage input = 2% × 2^(Ct 2% input sample − Ct IP sample)^, where Ct = threshold cycle of the PCR reaction.

### Western blotting.

Cells and tumor tissues were harvested and lysed with ice-cold RIPA lysis buffer (Thermo Fisher Scientific), and the Pierce BCA Protein Assay Kit was used to measure the concentration of proteins. Western blotting was performed as described previously (68) and the antibodies are listed in Supplemental Table 9. See complete unedited blots in the supplemental material.

### Cell invasion and migration assays.

Cell invasion and migration in vitro were determined using 8 μm pore size Transwell chambers (Corning Costar) with or without Matrigel (BioCoat) diluted 3:1 using PBS. HCT116 cells (2 × 10^4^) were seeded on the top chamber with 100 μL serum-free medium, and the conditioned medium of LX-2 cells was added into the bottom wells. Both the invaded and migrated cells were fixed with 4% paraformaldehyde for 30 minutes, stained with crystal violet solution (Sigma-Aldrich), and imaged under the Olympus BX 53 microscope. To evaluate the migration of human MDSCs, MDSCs (1 × 10^5^) were plated in the upper chambers and different conditioned medium was added in the lower chamber. After incubation for 6 hours, the number of MDSCs in the bottom compartments was counted as described previously (69).

### Determination of the intracellular Ca^2+^ mobilization.

HCT116 cells (1 × 10^5^ cells/well) or MDSCs (1 × 10^5^ cells/well) were seeded in black 96-well clear-bottomed plates for 24 hours prior to the experiment. The intracellular Ca^2+^ levels were measured using a Screen Quest Fluo-8 No Wash Calcium Assay Kit (AAT Bioquest) according to the manufacturer’s instructions. Absorbance (Ex/Em: 485/535 nm) was determined at steady state. Different conditioned medium was added after the steady-state measurement to induce calcium release from intracellular sources and determine intracellular calcium levels (50 μL/well). Absorbance was monitored for 225 seconds, and several readings were obtained using an EnVision Multimode Plate Reader (PerkinElmer).

### Coculture system.

LX-2 cells were cocultured with LSECs derived from bevacizumab-sensitive or -resistant HCT116 CRCLM xenografts using 0.4 μm pore size Transwell chambers (Corning Costar) with LSECs culture in the upper chamber. LX-2 cells were added to the lower chambers of Transwell plates and cultured overnight until they grew to 50% confluence, and the cells were then cultured with HCT116^FGFBP1^ or HT29^shNC^ cell–conditioned medium for 24 hours.

### ELISA.

An FGFBP1 ELISA Kit (Boster) and CXCL5 ELISA Kit (Solarbio) were used to measure FGFBP1 or CXCL5 levels in the cell culture supernatant according to the manufacturers’ instructions.

### Annexin V/PI assay.

LX-2 cells treated with vehicle or Z-GP-DAVLBH (1 μM) for 24 hours were harvested and stained with an Annexin V–FITC/PI assay kit (Beyotime). The percentage of apoptotic cells was measured using a FACSCanto system (BD Biosciences).

### Cell viability assay.

LX-2 cells were seeded in 96-well plates at 5 × 10^3^ cells/100 μL medium. Following incubation with or without Z-GP-DAVLBH for 24 hours, the cell viability was assessed using a Cell Counting Kit-8 (Targetmol) assay according to the manufacturer’s instructions.

### Scoring of morphological response to therapy.

The contrast-enhanced CT scans of CRCLM patients preoperatively treated with Chemo or Chemo+Bev were available for the analysis of morphological response to therapy using a method based on previously published morphological response criteria (70). Morphological response to RHGP was calculated based on whether the lesion changed from a homogeneous, low-attenuation lesion with a thin, sharply defined tumor-liver interface or a moderate degree of heterogeneous attenuation and a moderately defined tumor-liver interface to a heterogeneous attenuation and a thick, poorly defined tumor-liver interface after treatment. Morphological response was scored independently by 3 observers using the same criteria.

### Scoring of pathological response to therapy.

For scoring of the pathological response of CRCLM patients with RHGP treated preoperatively with Chemo or Chemo+Bev from contrast-enhanced CT scans, the largest area of the tumor surface area was measured using the greatest diameter and the greatest perpendicular distance, while the reduction rate was calculated as (tumor area prior to treatment – tumor area following treatment)/tumor area prior to treatment. Pathological response was scored independently by 3 experienced pathologists using the above method.

### Statistics.

Data are presented as mean ± SEM. Differences between 2 groups were evaluated using the 2-tailed, unpaired *t* test, and differences between more than 2 groups were evaluated using 1-way ANOVA followed by Tukey’s post hoc test. Survival curves were constructed using the Kaplan-Meier method and analyzed by the log-rank test. All statistical analyses were performed using GraphPad Prism 7.0 software. A *P* value of less than 0.05 was considered significant.

### Study approval.

The human CRC liver metastasis tissue specimens and CT scan images used in this study were approved by the Clinical Ethics Committee of the First Affiliated Hospital of Jinan University, and written informed consent was received from participants prior to inclusion in the study. The animal experiments were approved by the Institute of Experimental Animal Ethics Committee of Jinan University.

### Data availability.

Proteome data are available via ProteomeXchange with identifier PXD030202. The raw RNA-seq data have been deposited in NCBI’s Gene Expression Omnibus (GEO) and are accessible through the GEO Series accession numbers GSE207976, GSE208084, and GSE208091.

## Author contributions

DZ, W Ye, and MC designed the study. MQ, SF, MC, MH, YL, QM, WL, WD, and W Yin carried out the experiments. MQ, SF, MC, MH, and X Li performed data analysis. JP, CJ, WH, LX, SQ, XC, and JH provided pathology review and assessment of clinical and preclinical samples. JH, QQ, LD, X Liu, YY, and CS helped in the experimental design and manuscript writing. MC, MQ, and SF wrote the manuscript. DZ, W Ye, MH, TL, and QQ revised the manuscript. The order of the co–first authors was determined on the basis of their efforts and contributions to the manuscript. All authors have seen and approved the final version of the manuscript.

## Supplementary Material

Supplemental data

Supplemental table 11

Supplemental table 12

Supplemental table 13

## Version 1 08/11/2022

In-Press Preview

## Version 2 10/03/2022

Electronic publication
