# Targeting FAPα-expressing hepatic stellate cells overcomes resistance to antiangiogenics in colorectal cancer liver metastasis models

**DOI:** 10.1172/JCI168771

**Published:** 2023-02-01

**Authors:** Ming Qi, Shuran Fan, Maohua Huang, Jinghua Pan, Yong Li, Qun Miao, Wenyu Lyu, Xiaobo Li, Lijuan Deng, Shenghui Qiu, Tongzheng Liu, Weiqing Deng, Xiaodong Chu, Chang Jiang, Wenzhuo He, Liangping Xia, Yunlong Yang, Jian Hong, Qi Qi, Wenqian Yin, Xiangning Liu, Changzheng Shi, Minfeng Chen, Wencai Ye, Dongmei Zhang

Original citation: *J Clin Invest*. 2022;132(19):1–18. https://doi.org/10.1172/JCI157399

Citation for this corrigendum: *J Clin Invest*. 2023;133(3):e168771. https://doi.org/10.1172/JCI168771

After the publication of this article, the authors became aware that the β-actin blot shown in Supplemental Figure 11A was incorrect. The supplemental file has been corrected.

The authors regret the error. 

## Supplementary Material

Supplemental data

## Version 1 02/01/2023

Electronic publication

