# Supplementary material for: Targeting FAPα-expressing hepatic stellate cells overcomes resistance to antiangiogenics in colorectal cancer liver metastasis models
Source: J Clin Invest. 2022 Oct 3;132(19):e157399. doi: 10.1172/JCI157399 (PMC9525122; doi:10.1172/JCI157399)

Supplemental Materials for

Targeting FAPα-expressing hepatic stellate cells overcomes resistance to anti-angiogenics in colorectal cancer liver metastasis models

Ming Qi[#], Shuran Fan[#], Maohua Huang[#], Jinghua Pan, Yong Li, Qun Miao, Wenyu Lyu, Xiaobo Li, Lijuan Deng, Shenghui Qiu, Tongzheng Liu, Weiqing Deng, Xiaodong Chu, Chang Jiang, Wenzhuo He, Liangping Xia, Yunlong Yang, Jian Hong, Qi Qi, Wenqian Yin, Xiangning Liu, Changzheng Shi, Minfeng Chen*, Wencai Ye*, Dongmei Zhang*

[#]These authors contributed equally to this work.

*Corresponding author

Dr. Dongmei Zhang, College of Pharmacy, Jinan University, 601 West Huangpu Avenue, Guangzhou, 510632, P. R. China. Phone: 86.208.522.2653; E-mail: dmzhang701@jnu.edu.cn.

Dr. Wencai Ye, College of Pharmacy, Jinan University, 601 West Huangpu Avenue, Guangzhou, 510632, P. R. China. Phone: 86.208.522.0004; E-mail: chywc@aliyun.com.

Dr. Minfeng Chen, College of Pharmacy, Jinan University, 601 West Huangpu Avenue, Guangzhou, 510632, P. R. China. Phone: 86.208.522.8916; E-mail: minfengchen@jnu.edu.cn.

This PDF file includes:

Supplemental Figure 1 to 16
Supplemental Table 1 to 10

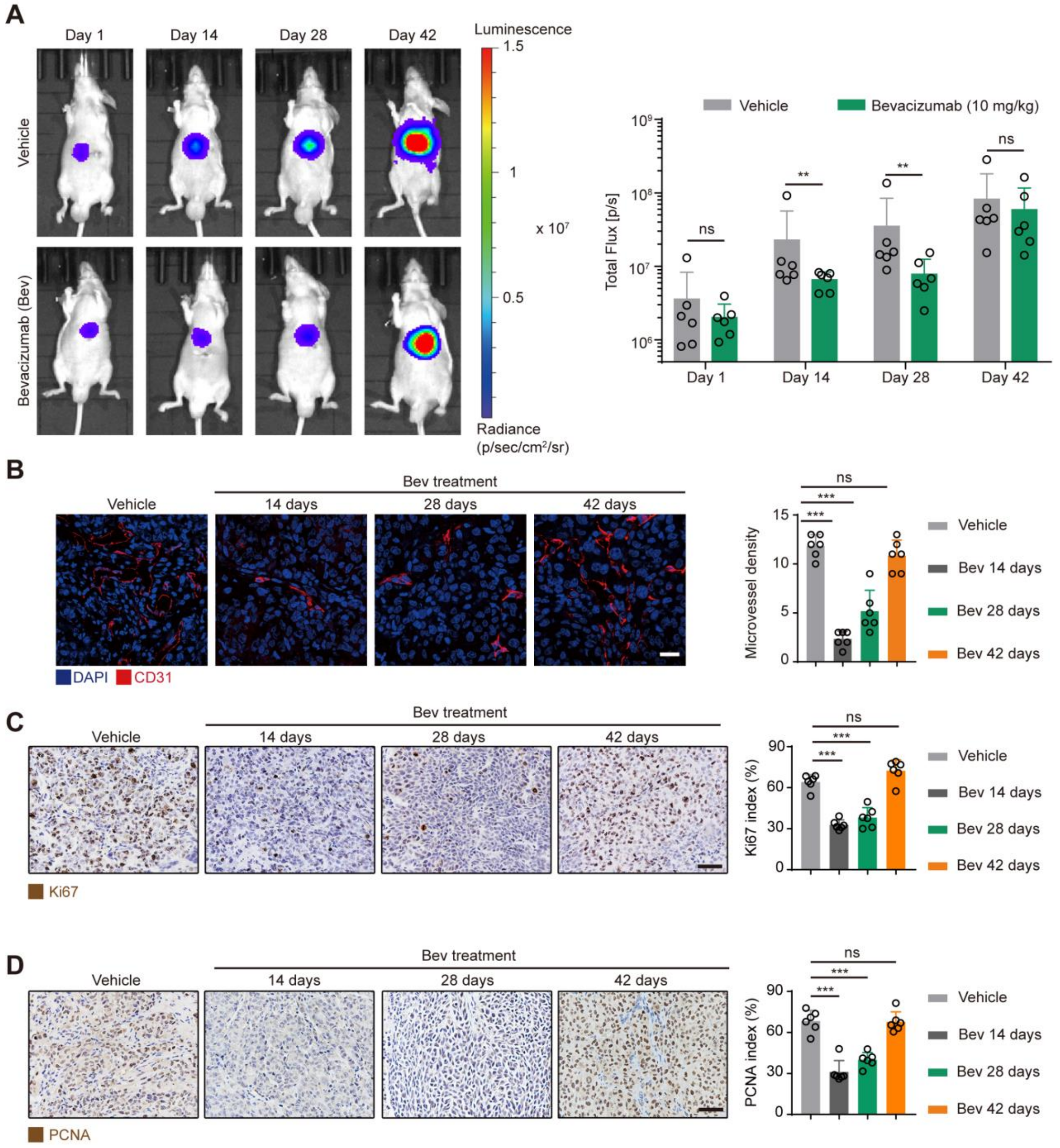

Supplemental Figure 1. Construction of the bevacizumab acquired resistant HCT116 CRCLM xenografts. (A) Representative images and quantification of bioluminescence signals in mice injected with HCT116-luc cells into the left main lobe of the liver (n = 6). (B) Immunofluorescence staining and quantification of CD31$^+$ (red) tumor vessels (n = 6). Scale bar, 20 μm. (C) Immunohistochemical staining and quantification of Ki67 in tumor tissues (n = 6). Scale bar, 50 μm. (D) Immunohistochemical staining and quantification of PCNA in tumor tissues (n = 6).

Scale bar, 50 μm. Data are presented as mean ± SEM. ns, no significance; $^{**}P < 0.01$, $^{***}P < 0.001$ (2-tailed unpaired *t* test in A; 1-way ANOVA with Tukey's post hoc comparison in B-D).

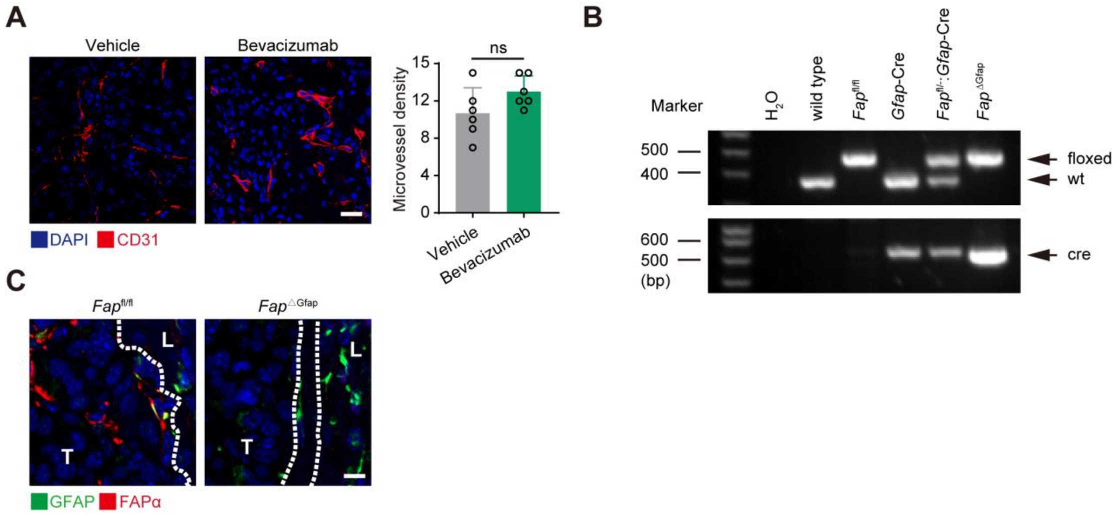

Supplemental Figure 2. Characteristics of the HSC-specific conditional *Fap*-knockout mice and the MC38 CRCLM allografts. (A) Immunofluorescence staining of $CD31^+$ tumor vessels (red) in MC38 CRCLM allografts. Quantification of microvessel density is shown (n = 6). Scale bar, 20 μm. (B) Genotyping of the transgenic mice. The genomic DNA derived from the indicated mice were analyzed by PCR assay, and the DNA fragments were separated on 2% agarose gels (n = 6). (C) Immunofluorescence staining of FAPα (red) in the $GFAP^+$ HSCs (green) in MC38 CRCLM allografts (n = 6). Scale bar, 10 μm. Data are presented as mean ± SEM. ns, no significance.

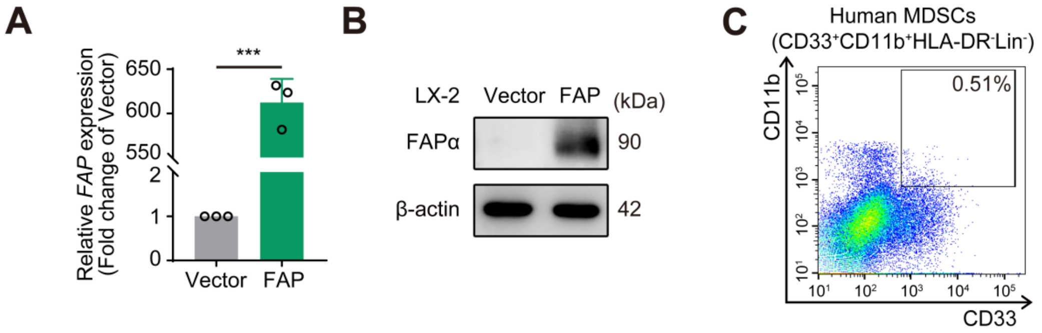

Supplemental Figure 3. FAPα^{+} HSCs facilitate vessel co-option via promoting recruitment of MDSCs and tumor cell EMT. (A) RT-qPCR analysis of the levels of *FAP* in LX-2 cells (n = 3). (B) Western blotting analysis of the expression of FAPα in LX-2 cells. (C) FCM analysis of MDSCs isolated from human CRC tumor samples. Data are presented as mean ± SEM. ***P < 0.001 (2-tailed unpaired t test).

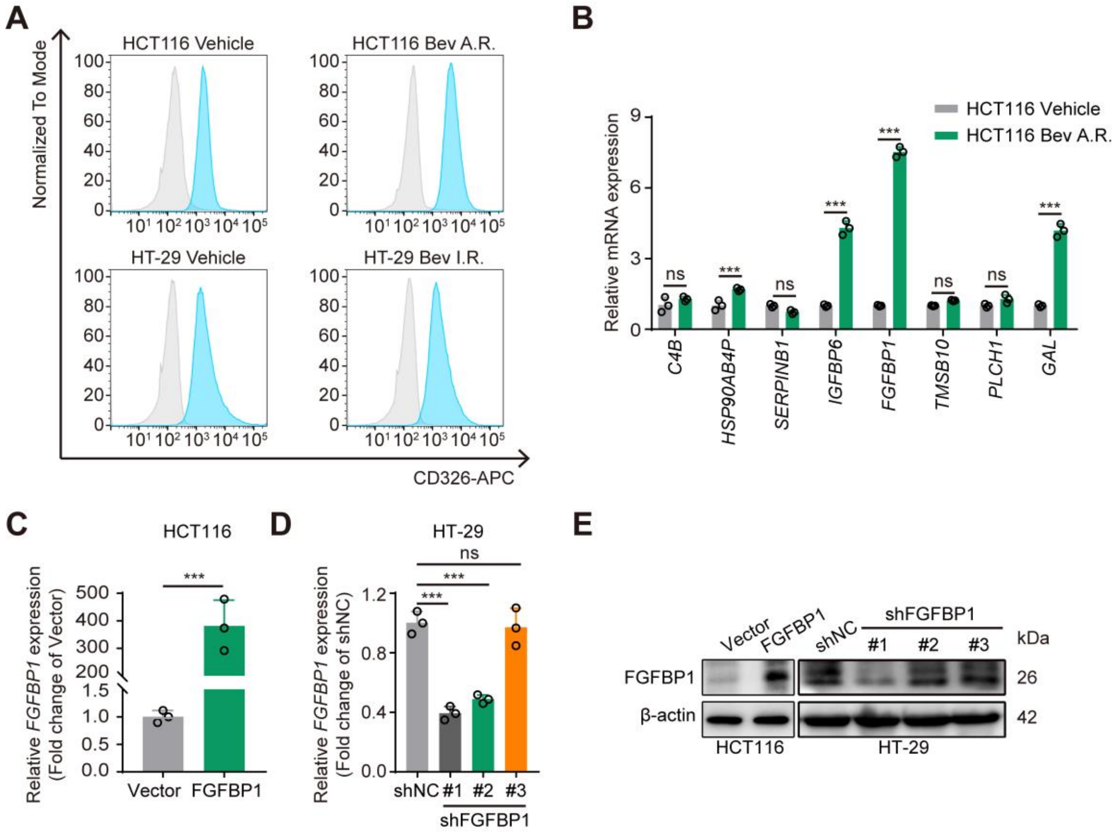

Supplemental Figure 4. Evaluation of the expression of FGFBP1 in HCT116 or HT-29 cells. (A) FCM analysis of the primary tumor cells isolated from bevacizumab-

sensitive or -resistant CRCLM xenografts. (B) RT-qPCR analysis of the levels of genes indicated by proteomic assay in HCT116 cells (n = 3). (C) RT-qPCR analysis of the overexpression of *FGFBP1* in HCT116 cells (n = 3). (D) RT-qPCR analysis of the knockdown of *FGFBP1* in HT-29 cells (n = 3). (E) Western blotting analysis of FGFBP1 in HCT116 and HT-29 cells after the indicated transfections. Data are presented as mean ± SEM. ns, no significance, ***P < 0.001 (2-tailed unpaired t test in B, C; 1-way ANOVA with Tukey's post hoc comparison in D).

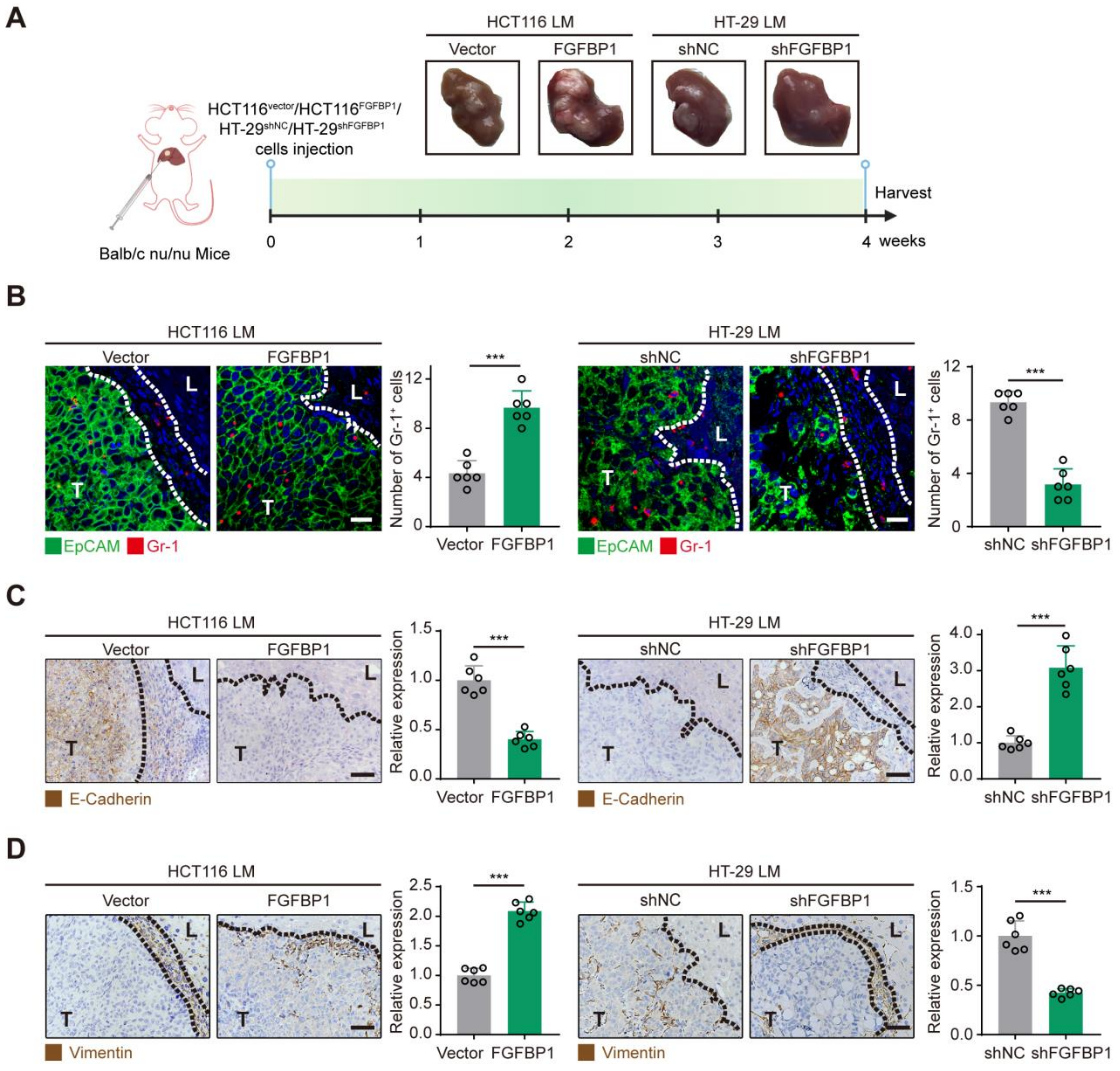

Supplemental Figure 5. Tumor-derived FGFBP1 promotes of MDSC recruitment and tumor cell EMT in CRCLM xenografts. (A) Schematic showing the

establishment of the FGFBP1-knockdown or -overexpression CRCLM xenografts. Tumors were harvested and photographed at the end of experiments (n = 6). (B) Immunofluorescence staining of EpCAM+ (green) tumor cells and Gr-1+ MDSCs (red) in the tumor-liver interface of CRCLM xenografts. Scale bar, 50 μm. Quantification of Gr-1+ MDSCs is shown (n = 6). (C) Immunohistochemical staining and quantification of E-cadherin in the tumor-liver interface of CRCLM xenografts (n = 6). Scale bar, 50 μm. (D) Immunohistochemical staining and quantification of vimentin in the tumor-liver interface of CRCLM xenografts (n = 6). Scale bar, 50 μm. Dotted lines indicate the tumor-liver interface. LM, liver metastases; T, Tumor; L, Liver. Data are presented as mean ± SEM. ***P < 0.001 (2-tailed unpaired t test).

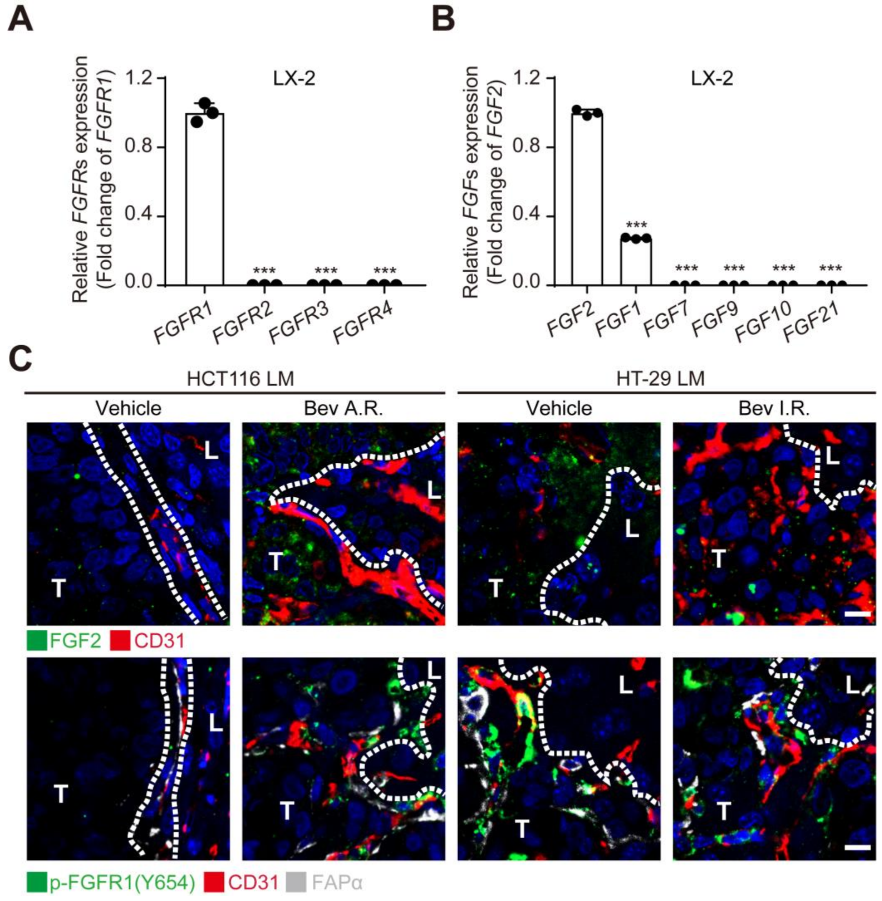

Supplemental Figure 6. FGF2 and FGFR1 are highly expressed in HSCs. (A) RT-qPCR assay analysis of the levels of *FGFR*s in LX-2 cells (n = 3). (B) RT-qPCR assay

analysis of the levels of *FGF*s in LX-2 cells (n = 3). (C) Immunofluorescence staining of FGF2^{+} (green), p-FGFR1^{+} (green), or FAPα^{+} (gray) HSCs, and CD31^{+} sinusoidal blood vessels (red) in the tumor-liver interface of CRCLM xenografts (n = 6). Scale bar, 10 µm. Data are presented as mean ± SEM. ***P < 0.001 compared to *FGFR1* or *FGF1* group (1-way ANOVA with Tukey's post hoc comparison).

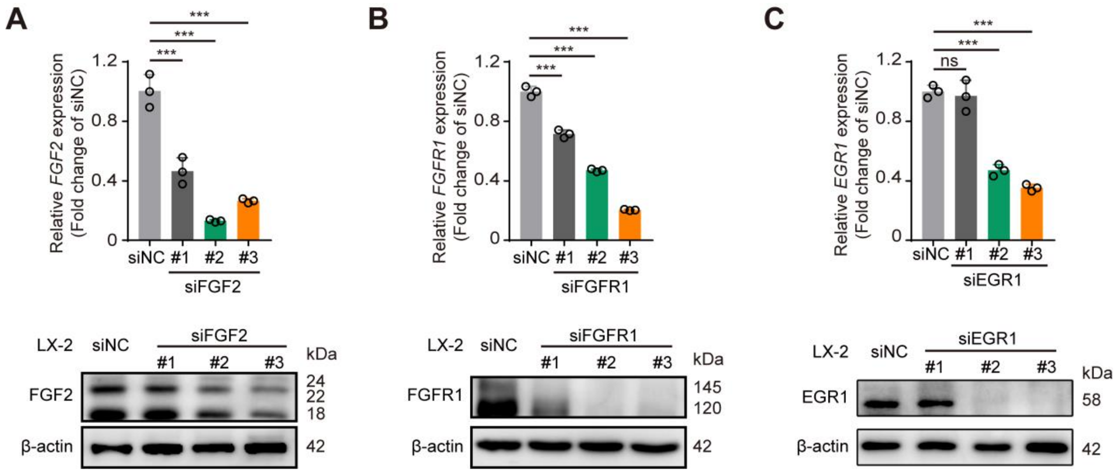

Supplemental Figure 7. Validation of knockdown efficiency of FGF2, FGFR1, and EGR1 in HSCs. (A-C) RT-qPCR and Western blotting analysis of the knockdown of FGF2 (A), FGFR1 (B), and EGR1 (C) in LX-2 cells (n = 3). Data are presented as mean ± SEM. ns, no significance, ***P < 0.001 (1-way ANOVA with Tukey's post hoc comparison).

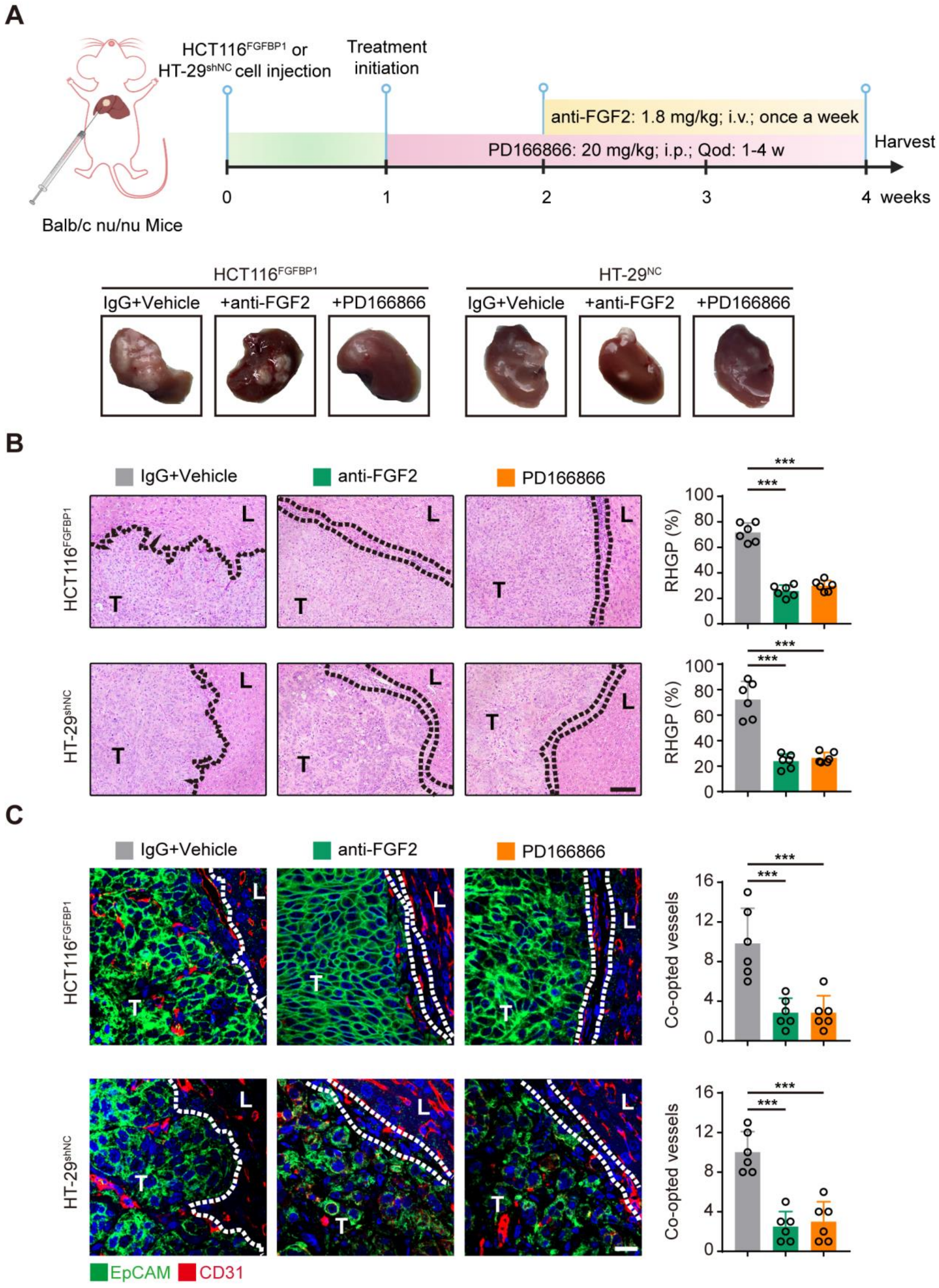

Supplemental Figure 8. Evaluation of the efficacy of FGF2 neutralizing antibody or PD166866 in FGFBP1-overexpressing CRCLM xenografts. (A) Experimental design of the FGF2 neutralizing antibody and PD-166866 (an FGFR1 inhibitor) treatments in HCT116 or HT-29 CRCLM xenografts (n = 6). (B) H&E staining of the

tumor-liver interface of CRCLM xenografts. Scale bar: 100 μm. Quantification of RHGP is shown (n = 6). (C) Immunofluorescence staining of the $EpCAM^+$ tumor cells (green) infiltrated into the liver parenchyma and hijacked the $CD31^+$ sinusoidal blood vessels (red) in the tumor-liver interface of CRCLM xenografts. Scale bar: 20 μm. Quantification of the co-opted sinusoidal blood vessels is shown (n = 6). Data are presented as mean ± SEM. ***$P < 0.001$(1-way ANOVA with Tukey's post hoc comparison).

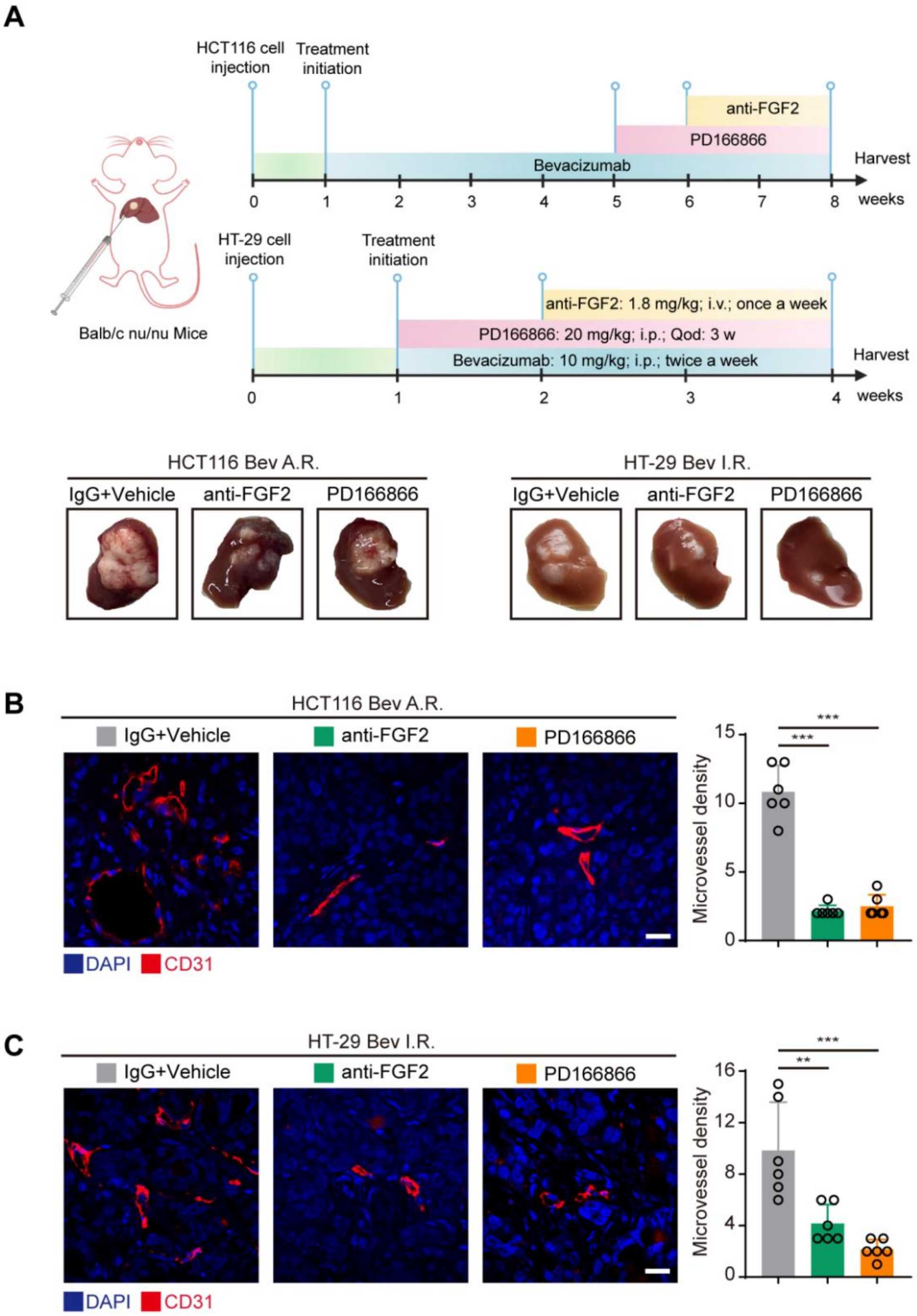

Supplemental Figure 9. Evaluation of the efficacy of FGF2 neutralizing antibody or PD166866 in bevacizumab-resistant CRCLM xenografts. (A) Therapeutic schedule for HCT116 and HT-29 xenografts treated with bevacizumab with or without FGF2 neutralizing antibody or PD166866 (n = 6). (B) Immunofluorescence staining and quantification of $CD31^+$ tumor vessels (red) in HCT116 CRCLM xenografts (n = 6). Scale bar, 20 µm. (C) Immunofluorescence staining and quantification of $CD31^+$

tumor vessels (red) in HT-29 CRCLM xenografts (n = 6). Scale bar, 20 μm. Data are presented as mean ± SEM. $^{**}P < 0.01$, $^{***}P < 0.001$ (1-way ANOVA with Tukey's post hoc comparison).

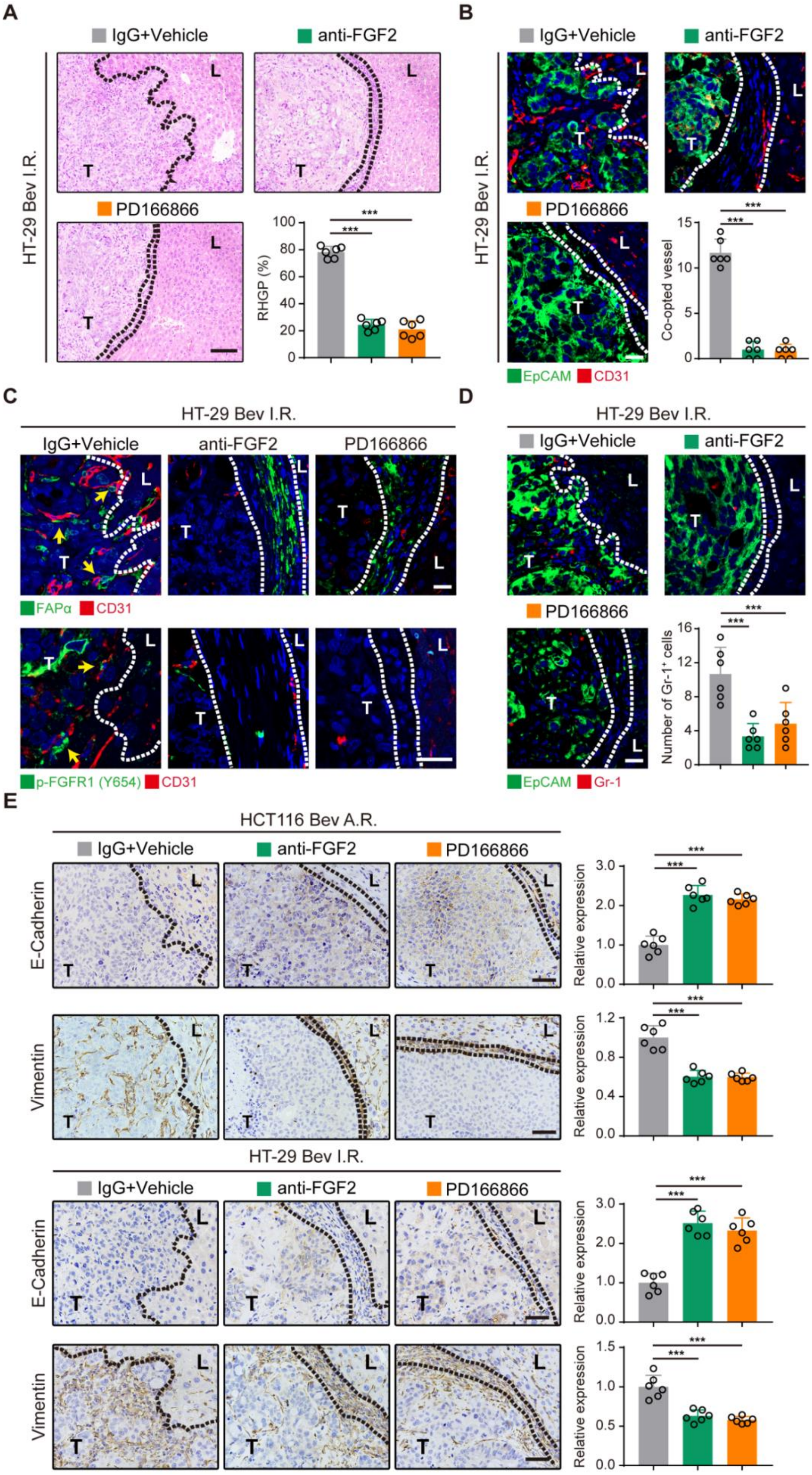

Supplemental Figure 10. Blockade of FGF2-FGFR1 signaling pathway in HSCs attenuates the bevacizumab-induced vessel co-option. (A) H&E staining of the

tumor-liver interface of HT-29 CRCLM xenografts. Scale bar: 100 μm. Quantification of RHGP is shown (n = 6). (B) Immunofluorescence staining of the EpCAM$^+$ tumor cells (green) infiltrated into the liver parenchyma and hijacked the CD31$^+$ sinusoidal blood vessels (red) in the tumor-liver interface of HT-29 CRCLM xenografts. Scale bar: 20 μm. Quantification of the co-opted sinusoidal blood vessels is shown (n = 6). (C) Immunofluorescence staining of the FAPα (green) and p-FGFR1$^+$ in HSCs attached on the CD31$^+$ sinusoidal blood vessels (red) in the tumor-liver interface of HT-29 CRCLM xenografts. Scale bar, 20 μm. Yellow arrows indicate the FAPα$^+$ or p-FGFR1$^+$ HSCs. (D) Immunofluorescence staining of the EpCAM$^+$ (green) tumor cells and Gr-1$^+$ MDSCs (red) in the tumor-liver interface of HT-29 CRCLM xenografts. Scale bar, 20 μm. Quantification of Gr-1$^+$ MDSCs is shown (n = 6). (E) Immunohistochemical staining and quantification of E-cadherin and vimentin in the tumor-liver interface of HCT116 and HT-29 CRCLM xenografts (n = 6). Scale bar, 50 μm. Dotted lines indicate the tumor-liver interface. Bev I.R., bevacizumab intrinsic resistance. T, Tumor. L, Liver. Data are presented as mean ± SEM. ***$P < 0.001$ (1-way ANOVA with Tukey's post hoc comparison).

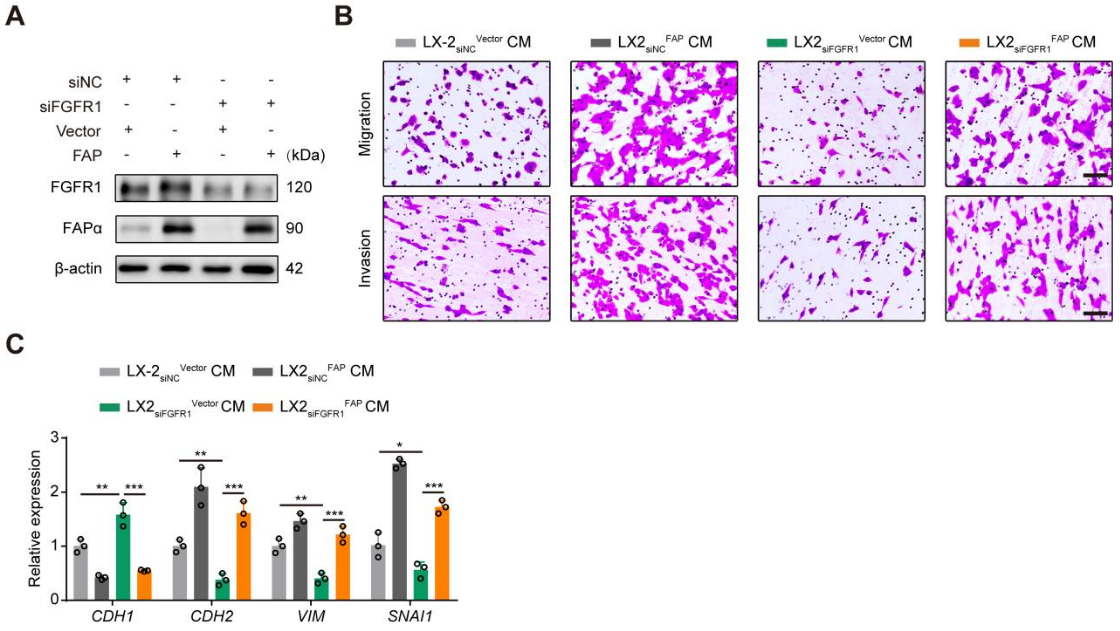

Supplemental Figure 11. FGFR1-FAPα axis is essential for tumor cell EMT. (A) Western blotting analysis of FGFR1 and FAPα in LX-2 cells with FGFR1-knockdown and/or FAPα-overexpression. (B) Transwell assays of the migration and invasion of HCT116 cells treated with the conditioned medium from LX-2 cells (n = 3). (C) RT-qPCR analysis of *CDH1*, *CDH2*, *SNAI1*, and *VIM* in HCT116 cells treated with the conditioned medium from LX-2 cells (n = 3). Data are presented as mean ± SEM. ns, no significance; *$P < 0.05$, **$P < 0.01$, and ***$P < 0.001$ (1-way ANOVA with Tukey's post hoc comparison). CM: conditioned medium.

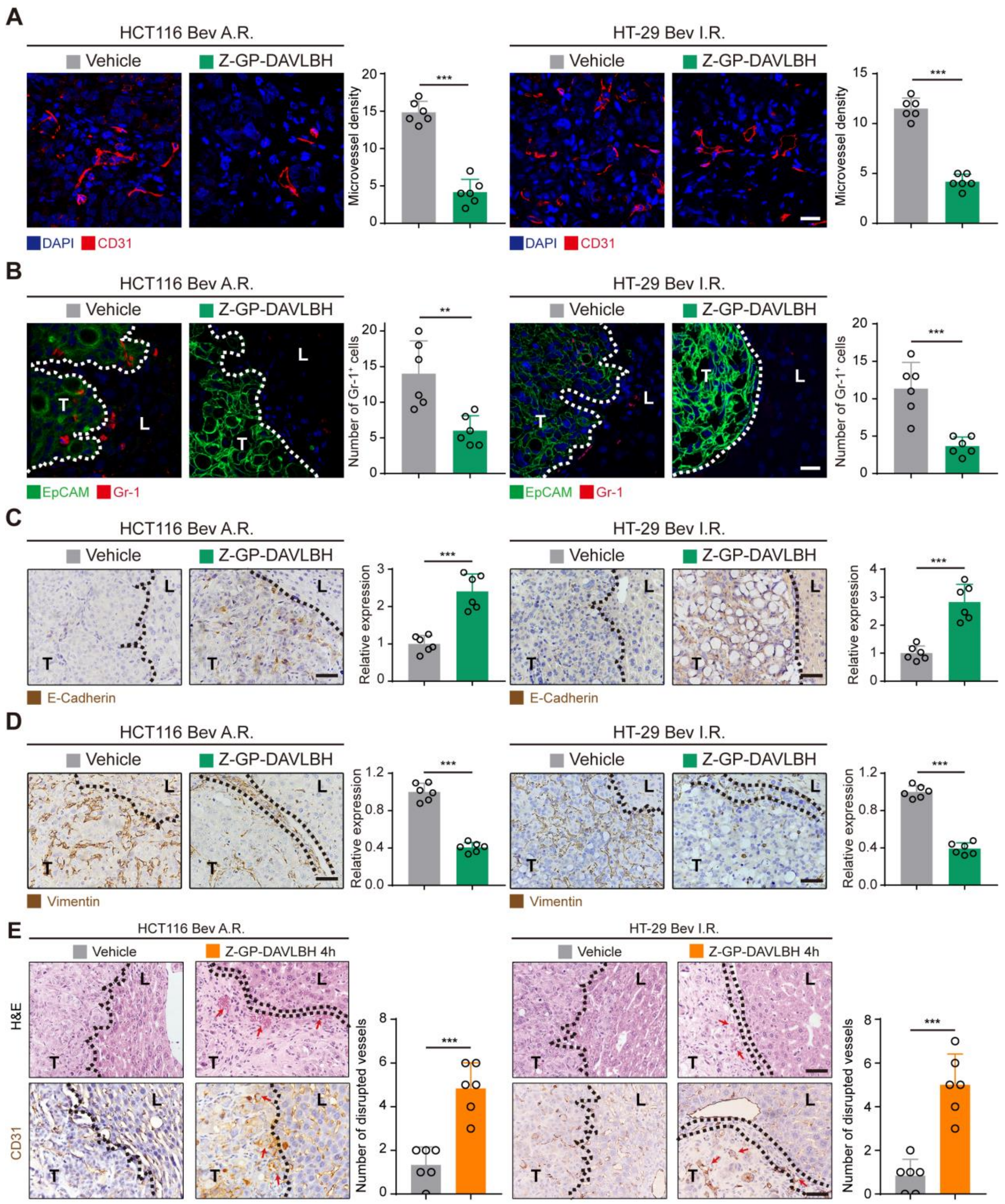

Supplemental Figure 12. Z-GP-DAVLBH inhibits the recruitment of MDSCs and tumor cell EMT. (A) Immunofluorescence staining and quantification of CD31$^+$ tumor vessels (red) in HCT116 and HT-29 CRCLM xenografts (n = 6). Scale bar, 20 µm. (B) Immunofluorescence staining of Gr-1$^+$ MDSCs (red) and EpCAM$^+$ tumor cells (green) in the liver-tumor interface of CRCLM xenografts. Scale bar, 20 µm. Quantification of Gr-1$^+$ MDSCs is shown (n = 6). (C) Immunohistochemical staining and quantification of E-cadherin in the liver-tumor interface of CRCLM xenografts (n = 6). Scale bar, 50

μm. (D) Immunohistochemical staining and quantification of vimentin in the tumor-liver interface of CRCLM xenografts (n = 6). Scale bar, 50 μm. (E) H&E and immunohistochemical staining showed that Z-GP-DAVLBH induced vessel disruption in the tumor-liver interface of CRCLM xenografts. Red arrows indicate the "hole-like" disruptions in the tumor vessels. Scale bar, 50 μm. Quantification of the disrupted vessels is shown (n = 6). T, Tumor. L, Liver. Data are presented as mean ± SEM. $^{**}P < 0.01$, $^{***}P < 0.001$ (2-tailed unpaired *t* test).

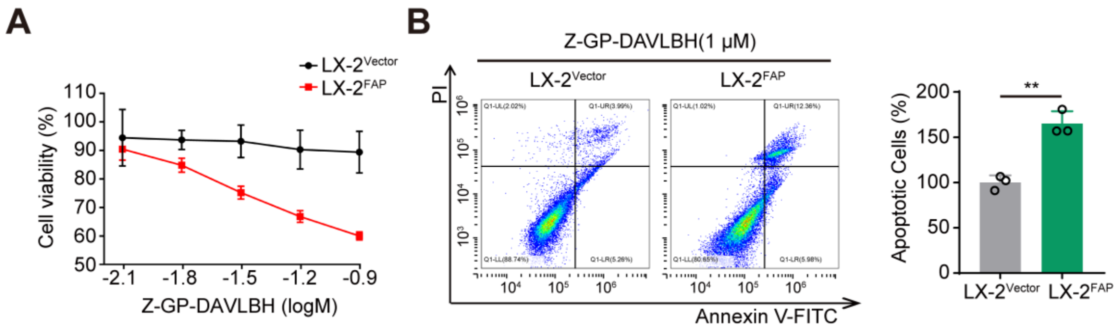

Supplemental Figure 13. Z-GP-DAVLBH selectively induces apoptosis in FAPα⁺ HSCs. (A) CCK-8 assay analysis of the effect of Z-GP-DAVLBH on the cell viability of LX-2 cells (n = 3). (B) FCM analysis of the apoptotic LX-2 cells after treatment with Z-GP-DAVLBH (1 μM) for 4 h (n = 3). Data are presented as mean ± SEM. $^{**}P < 0.01$ (2-tailed unpaired *t* test).

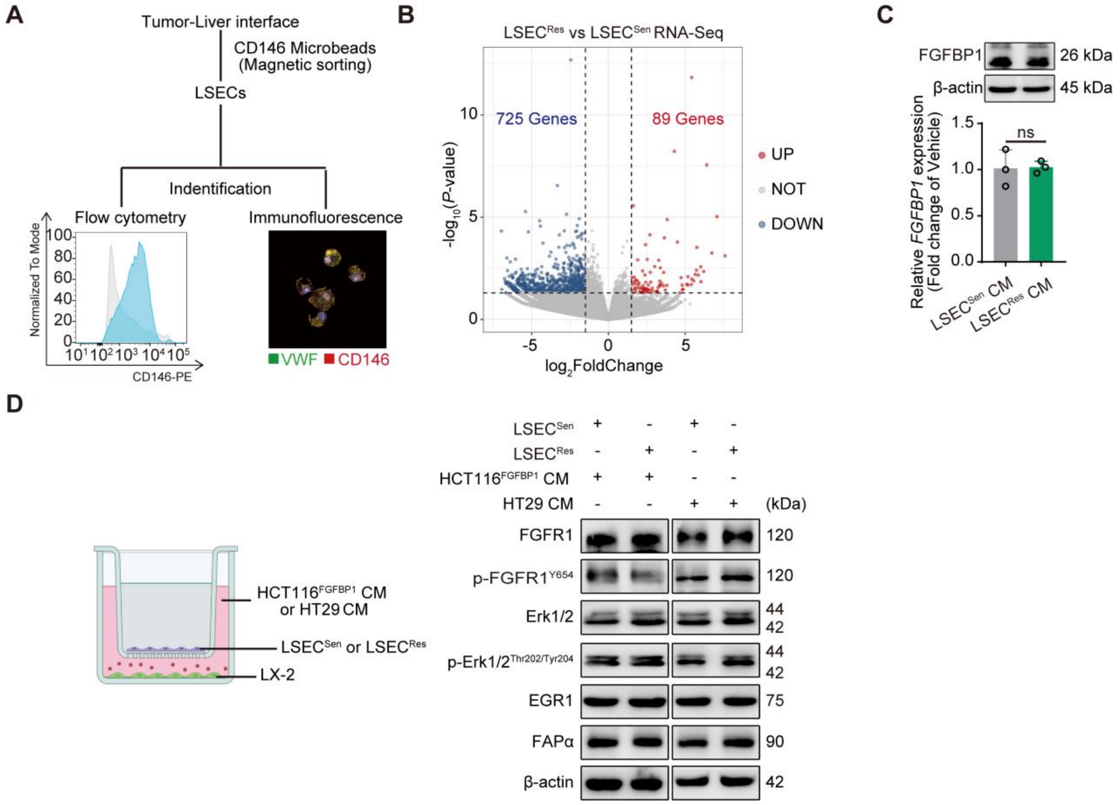

Supplemental Figure 14. Effects of liver sinusoidal endothelial cells on LX2 cells and HCT116 cells. (A) Schematic diagram of the isolation and identification of primary LSECs from the liver-tumor interface of HCT116 CRCLM xenografts. (B) Volcano plot depicting the differentially expressed genes in LSECs obtained from bevacizumab-sensitive and -resistant HCT116 CRCLM xenografts (log2FoldChange > 1.5, P-value < 0.05; n = 3). Res, bevacizumab-resistant. Sen, bevacizumab-sensitive. (C) RT-qPCR and Western blotting analysis of FGFBP1 in HCT116 cells treated with the conditioned medium from LSECs (n = 3). (D) Western blotting analysis of FGFR1, p-FGFR1, ERK1/2, p-ERK1/2, EGR1, and FAPα in LX-2 cells cocultured with LSECs and treated with the conditioned medium from HCT116 or HT-29 cells. Data are presented as mean ± SEM. ns, no significance (2-tailed unpaired t test). CM: conditioned medium.

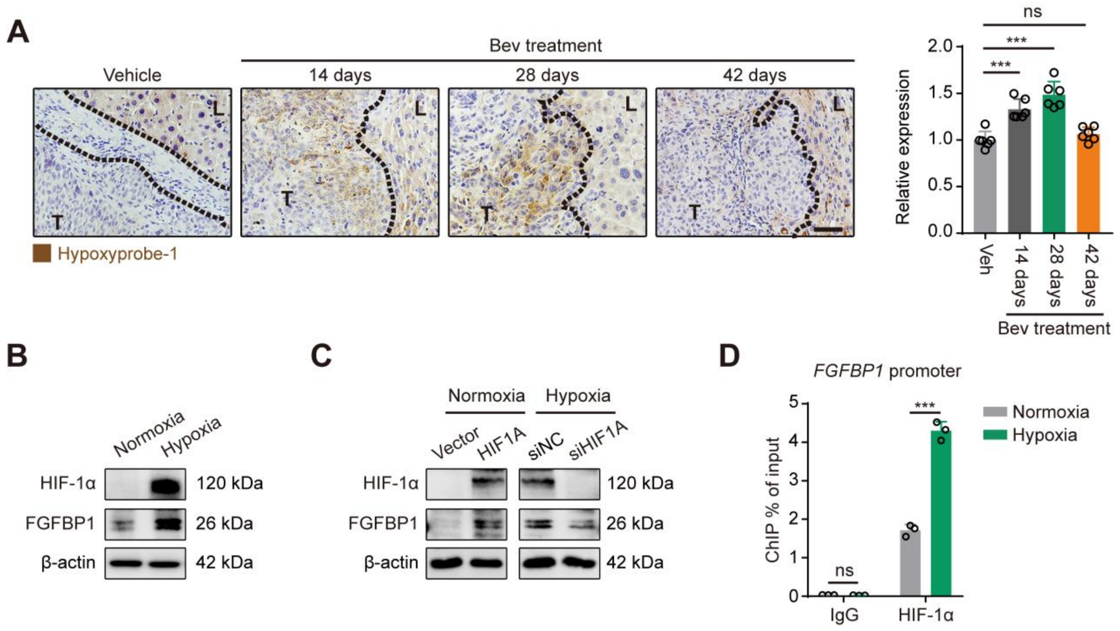

Supplemental Figure 15. Hypoxia is responsible for the expression of FGFBP1 in tumor cells. (A) Immunohistochemical staining and quantification of hypoxyprobe-1 in bevacizumab-treated HCT116 CRCLM xenografts (n = 6). Scale bar, 50 μm. (B) Western blotting analysis of HIF-1α and FGFBP1 in normoxic or hypoxic HCT116 cells. (C) Western blotting analysis of HIF-1α and FGFBP1 in normoxic or hypoxic HCT116 cells with HIF1α-overexpression or -knockdown. (D) ChIP-qPCR analysis of the binding of HIF-1α to the *FGFBP1* promoter (n = 3). Data are presented as mean ± SEM. ns, no significance; ****P* < 0.001 (1-way ANOVA with Tukey's post hoc comparison in A; 2-tailed unpaired *t* test in D).

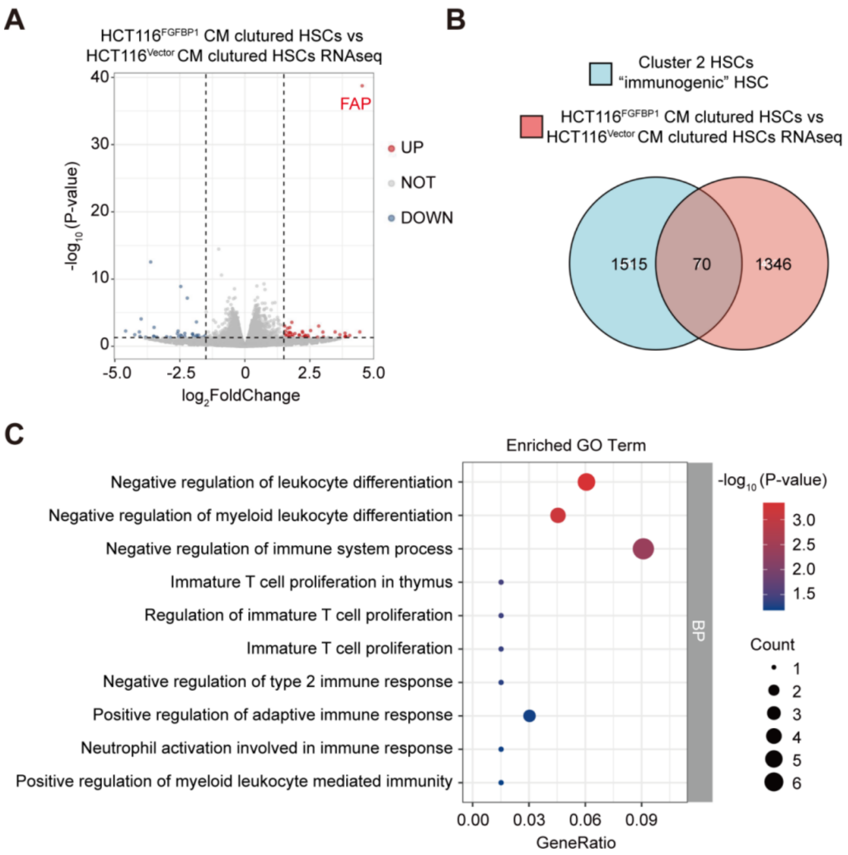

Supplemental Figure 16. HSCs may partially transform into an "immunogenic" phenotype during vessel co-option. (A) Volcano Plot depicting differentially expressed genes in LX-2 cells primed with the conditioned medium from HCT116 cells (log2FoldChange > 1.5, *P*-value < 0.05; n = 3). (B) Venn diagram illustrating the overlapping genes between the upregulated genes in HCT116 cell conditioned medium-treated LX-2 cells analyzed by RNA-seq and the differential expression genes (DEG) in cluster 4 HSCs ("immunogenic" HSCs) analyzed by scRNA-seq (GSE171904). (C) Gene ontology (GO) enrichment analysis of the 70 overlapping genes in (B).

Supplemental Table 1. Characteristics of 45 patients (n = 53 lesions) treated preoperatively with Chemo or Chemo+Bev prior to liver resection at Guangzhou Overseas Chinese Hospital (Detailed patients' information in Figure 7A).

**Demographics**	
Gender, number of patients (%)	
Male	32 (71.1)
Female	13 (28.9)
Age, median (range)	56 (27 – 75)
**Primary tumor**	
Site of primary tumor, number of patients (%)	
Rectum	24 (53.3)
Recto–sigmoid	11 (24.5)
Colon	10 (22.2)
Lymph node status, number of patients (%)	
Positive	28 (62.2)
Negative	17 (37.8)
Histological grade, number of patients (%)	
High grade	1 (2.2)
Moderate grade	42 (93.3)
Low grade	2 (4.5)
Adjuvant Bev therapy, number of patients (%)	
Yes	31 (68.9)
No	14 (31.1)
**Liver metastasis**	
No. of liver lesions at presentation, number of patients (%)	
Solitary lesion	40 (88.9)
Multiple lesions	5 (11.1)
Preoperative therapy administered, number of patients (%)	
CAPOX + bevacizumab	4 (8.9)
FOLFOX + bevacizumab	14 (31.1)
FOLFIRI + bevacizumab	9 (20.0)
FOLFOXIRI+ bevacizumab	3 (6.7)
Oxaliplatin+Capecitabine+Bevacizumab	1 (2.2)
FOLFOX	7 (15.5)
FOLFOXIRI	3 (6.7)
CAPOX	4 (8.9)

Footnote: CAPOX, capecitabine and oxaliplatin; FOLFOX, infusional 5-fluorouracil and oxaliplatin; FOLFIRI, infusional 5-fluorouracil and irinotecan; FOLFOXIRI, infusional 5-fluorouracil, oxaliplatin and irinotecan. See Supplemental Table 11 for further detailed information.

Supplemental Table 2. Characteristics of 66 patients (n = 82 lesions) treated preoperatively with Chemo or Chemo+Bev prior to liver resection at Guangzhou Overseas Chinese Hospital (Detailed patients' information in Figure 7, B-E).

**Demographics**	
Gender, number of patients (%)	
Male	45 (68.2)
Female	21 (31.8)
Age, median (range)	56 (27 – 75)
**Primary tumor**	
Site of primary tumor, number of patients (%)	
Rectum	28 (42.4)
Recto–sigmoid	21 (31.8)
Colon	16(24.3)
Cecum	1(1.5)
Lymph node status, number of patients (%)	
Positive	41 (62.1)
Negative	25 (37.9)
Histological grade, number of patients (%)	
High grade	3 (4.5)
Moderate grade	59 (89.4)
Low grade	4 (6.1)
Adjuvant Bev therapy, number of patients (%)	
Yes	37 (56.1)
No	29 (43.9)
**Liver metastasis**	
No. of liver lesions at presentation, number of patients (%)	
Solitary lesion	54 (81.8)
Multiple lesions	12 (18.2)
Preoperative therapy administered, number of patients (%)	
CAPOX + bevacizumab	11 (16.7)
FOLFOX + bevacizumab	15 (22.7)
FOLFIRI + bevacizumab	8 (12.1)
FOLFOXIRI+ bevacizumab	3 (4.5)
CAPOX	10 (15.2)
FOLFOX	12 (18.2)
FOLFIRI	2(3.0)
FOLFOXIRI	5 (7.6)

Footnote: CAPOX, capecitabine and oxaliplatin; FOLFOX, infusional 5-fluorouracil and oxaliplatin; FOLFIRI, infusional 5-fluorouracil and irinotecan; FOLFOXIRI, infusional 5-fluorouracil, oxaliplatin and irinotecan. See Supplemental Table 12 for further detailed information.

Supplemental Table 3. Characteristics of 34 patients (n = 42 lesions) treated preoperatively with Chemo or Chemo+Bev prior to liver resection at Guangzhou Overseas Chinese Hospital (Detailed patients' information in Figure 7F).

**Demographics**	
Gender, number of patients (%)	
Male	22 (64.7)
Female	12 (35.3)
Age, median (range)	55 (28 – 73)
**Primary tumor**	
Site of primary tumor, number of patients (%)	
Rectum	11 (32.3)
Recto–sigmoid	14 (41.2)
Colon	8(23.5)
Cecum	1（3.0）
Lymph node status, number of patients (%)	
Positive	21 (61.8)
Negative	13 (38.2)
Histological grade, number of patients (%)	
High grade	2 (5.9)
Moderate grade	30 (88.2)
Low grade	2 (5.9)
Adjuvant Bev therapy, number of patients (%)	
Yes	18 (52.9)
No	16 (47.1)
**Liver metastasis**	
No. of liver lesions at presentation, number of patients (%)	
Solitary lesion	28 (82.4)
Multiple lesions	6 (17.6)
Preoperative therapy administered, number of patients (%)	
FOLFOX + bevacizumab	7 (20.6)
FOLFIRI + bevacizumab	3 (8.8)
FOLFOXIRI+ bevacizumab	1 (3.0)
CAPOX+ bevacizumab	6 (17.6)
Oxaliplatin+Capecitabine+Bevacizumab	1 (3.0)
FOLFOX	6 (17.6)
FOLFOXIRI	3 (8.8)
CAPOX	7 (20.6)

Footnote: CAPOX, capecitabine and oxaliplatin; FOLFOX, infusional 5-fluorouracil and oxaliplatin; FOLFIRI, infusional 5-fluorouracil and irinotecan; FOLFOXIRI, infusional 5-fluorouracil, oxaliplatin and irinotecan. See Supplemental Table 13 for further detailed information.

Supplemental Table 4. List of primers sequences for PCR analysis of genomic DNA from tail snips.

Primer name	Sequence	Band Size
T004857-F1A	GGGCAGTCTGGTACTTCCAAGCT	WT:0bp
T004857-R1A	GGGCAGUAUAAACUUGGAUTT	Targeted:515bp
T052266-F1	CATCGCATTGTCTGAGTAGGTG	WT:365bp
T052266-R1	CTCCAGCAAGGCTGCACAAT	Targeted:467bp

Supplemental Table 5. List of shRNA sequences for cell transfection.

Gene name	Target sequence
FGFBP1-shRNA-1	GAGGGCATCTCTCTCAAGGTT
FGFBP1-shRNA-2	AAGCTAGTCAGCTCCACTCTA
FGFBP1-shRNA-3	CAGGGAGCACATCAAAGGCAA

Supplemental Table 6. List of siRNA sequences for cell transfection.

Gene name	Sense sequence (5' to 3')	Antisense sequence (5' to 3')
FGF2-Homo-638	CCCUCACAUCAAGCUACAATT	UUGUAGCUUGAUGUGAGGGTT
FGF2-Homo-693	GGAGUGUGUGCUAACCGUUTT	AACGGUUAGCACACACUCCTT
FGF2-Homo-858	GGGCAGUAUAAACUUGGAUTT	AUCCAAGUUUAUACUGCCCTT
FGFR1-Homo-1265	GCAGUGACACCACCUACUUTT	AAGUAGGUGGUGUCACUGCTT
FGFR1-Homo-2598	GCAGGAUGGUCCCUUGUAUTT	AUACAAGGGACCAUCCUGCTT
FGFR1-Homo-3116	GCACCAACGAGCUGUACAUTT	AUGUACAGCUCGUUGGUGCTT
EGR1-Homo-1354	CCCGGUUACUACCUCUUAUTT	AUAAGAGGUAGUAACCGGGTT
EGR1-Homo-1264	GGACAAGAAAGCAGACAAATT	UUUGUCUGCUUUCUUGUCCTT
EGR1-Homo-324	CUCUGAACAACGAGAAGGUTT	ACCUUCUCGUUGUUCAGAGTT
HIF1A -Homo-964	GCUGAUUUGUGAACCCAUUTT	AAUGGGUUCACAAAUCAGCTT

Supplemental Table 7. List of antibodies for immunohistochemistry and immunofluorescence.

Antibodies	Supplier; catalogue number
Goat anti-CD31	R&D System; AF3628
Rabbit anti- CD31	Servicebio; GB14033
Rabbit anti-EpCAM	Abcam; ab71916
Sheep anti-FAPα	R&D System; AF3715
Rabbit anti- FAPα	Affinity; AF5344
Mouse anti-Gr-1	R&D System; MAB1037

Rabbit anti-CD8	Abcam; ab217344
Mouse anti-α-SMA	Servicebio; GB13044
Rabbit anti-GFAP	Servicebio; GB11096
Rabbit anti-CK18	Servicebio; GB11232
Mouse anti-E-Cadherin	Cell Signal Technology; 14472S
Mouse anti-Vimentin	Abcam; ab8978
Rabbit anti-N-cadherin	Proteintech; 22018-1-AP
Rabbit anti- FGFBP1	Proteintech; 25006-1-AP
Rabbit anti-EGR1	Cell Signal Technology; 4154s
Rabbit anti-Ki67	Servicebio; GB111141
Rabbit anti-PCNA	Servicebio; GB11010
Rabbit anti-p-FGFR1(phospho Y654)	Abcam; ab59194
Rabbit anti-FGF2	Beyotime; AF6891
Rabbit anti-Cleaved-PARP	Cell Signal Technology;94885S
anti-mouse IgG, HRP-linked Antibody	Cell Signal Technology; 2624S
anti-rabbit IgG, HRP-linked Antibody	Cell Signal Technology; 3670S
anti-goat IgG, HRP-linked Antibody	Servicebio; GB23204
anti-sheep IgG, HRP-linked Antibody	Abcam; ab6900
iF488-Tyramide	Servicebio; G1231
iF555-Tyramide	Servicebio; G1233
iF647-Tyramide	Servicebio; G1232
Alexa Fluor® 488 Goat anti-Rabbit	Invitrogen; A-11035

Supplemental Table 8. List of primer sequences for the RT-qPCR assay.

**Gene**	**Forward primer**	**Reverse primer**
CDH1	TCTCTCACGCTGTGTCATCC	CACTGGATTTGTGGTGACGA
CDH2	AGGCGTCTGTAGAGGCTTCTGG	TCTGCTGACTCCTTCACTGACTCC
VIM	GTCCGTGTCCTCGTCCTCCTAC	AGTTGGCGAAGCGGTCATTCAG
FAP	TACCCAAAGGCTGGAGCTAA	ACAGGACCGAAACATTCTGG
FGFBP1	TCTGGGCAACACCCAGAT	GGCATGAGGTTGGATTGC
SNAI1	CCTGGTCAAGAAGCATTTCAACGC	GGAGGAGGTGTCAGATGGAGGAG
FGF1	ACAGCCCTGACCGAGAAGTT	CCGTTGCTACAGTAGAGGAGT
FGF2	AGAAGAGCGACCCTCACATCA	CGGTTAGCACACACTCCTTTG
FGF7	TCCTGCCAACTTTGCTCTACA	CAGGGCTGGAACAGTTCACAT
FGF10	CAGTAGAAATCGGAGTTGTTGCC	TGAGCCATAGAGTTTCCCCTTC
FGF21	GCCTTGAAGCCGGGAGTTATT	GTGGAGCGATCCATACAGGG
FGFR1	GGCTACAAGGTCCGTTATGCC	GATGCTGCCGTACTCATTCTC
FGFR2	GGTGGCTGAAAAACGGGAAG	AGATGGGACCACACTTTCCATA

FGFR3	CCCAAATGGGAGCTGTCTCG	CCCGGTCCTTGTCAATGCC
FGFR4	GAGGGGCCGCCTAGAGATT	CAGGACGATCATGGAGCCT
IL1B	CCACAGACCTTCCAGGAGAATG	GTGCAGTTCAGTGATCGTACAGG
IL33	GCCTGTCAACAGCAGTCTACTG	TGTGCTTAGAGAAGCAAGATACTC
CSF2	GGAGCATGTGAATGCCATCCAG	CTGGAGGTCAAACATTTCTGAGAT
IL-18	GATAGCCAGCCTAGAGGTATGG	CCTTGATGTTATCAGGAGGATTCA
CXCL5	CAGACCACGCAAGGAGTTCATC	TTCCTTCCCGTTCTTCAGGGAG
EGR1	AGCAGCACCTTCAACCCTCAGG	GAGTGGTTTGGCTGGGGTAACT
ACTB	TCTTCCAGCCTTCCTTCCTG	CCTGCTTGCTGATCCACATC
C4B	AGATGCGGTGTCCAAGGTTCTG	GTTGCCAGGTATTTCCAAGGTCC
HSP90AB4P	GGAAGACCACTTGGCAGTCAAG	CAAAGCCGACATACTCTGGCATC
SERPINB1	AGCTCAGCATGGTCATCCTGCT	CGAGATTCTCAGGTTTAGTCCAC
IGFBP6	CACAGGATGTGAACCGCAGAGA	CACTGAGTCCAGATGTCTACGG
PLCH1	CTTTGGTTCGGTTCCTTGTGTGG	CTTTGGTTCGGTTCCTTGTGTGG
GAL	TCAGGTATGGACCTGTCAAAGCT	TGGCAACCACAGGTCATTCAGC
TMSB10	TGGCAGACAAACCAGACATGG	CGAAGAGGACGGGGGTAGG

Supplemental Table 9. List of antibodies for the Western blotting and ChIP assays.

Antibodies	Supplier; catalogue number
Sheep anti-FAPα	R&D System; AF3715
Mouse anti-β-actin	Cell Signal Technology; 3700S
Mouse anti-E-Cadherin	Cell Signal Technology; 14472S
Rabbit anti-N-Cadherin	Cell Signal Technology; 4061S
Mouse anti-Vimentin	Abcam; ab8978
Rabbit anti-Snail	Abcam; ab180714
Rabbit anti-FGFBP1	Proteintech;25006-1-AP
Rabbit anti-FGFR1	Cell Signal Technology; 9740s
Rabbit anti-p-FGFR1(phospho Y654)	Abcam; ab59194
Rabbit anti-FGF2	Beyotime; AF6891
Rabbit anti-EGR1	Cell Signal Technology; 4154s
Rabbit anti-Erk1/2	Cell Signal Technology; 4695s
Rabbit anti-p-Erk1/2 (Thr202/Tyr204)	Cell Signal Technology; 4370s
Rabbit anti-HIF1α	Cell Signal Technology; 36169s

anti-mouse IgG, HRP-linked Antibody	Cell Signal Technology; 2624s
anti-rabbit IgG, HRP-linked Antibody	Cell Signal Technology; 5127s
anti-sheep IgG, HRP-linked Antibody	Abcam; ab6900
anti-Rabbit lgG	Cell Signal Technology; 2729s

Supplemental Table 10. List of primer sequences for the ChIP-qPCR assay.

Gene promoter	Forward primer	Reverse primer
FAP	AGCACACCTTGGATATTACTC	ATGAGTCATTAGGCACTTAC
FGFBP1	GCCAGGAACTCTACCATAGGT	TTGGTGGTAAGGTTCACAGGT

Full unedited gel for Figure 3I

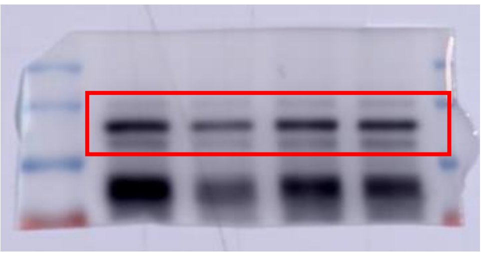

E-cadherin (97 kDa)

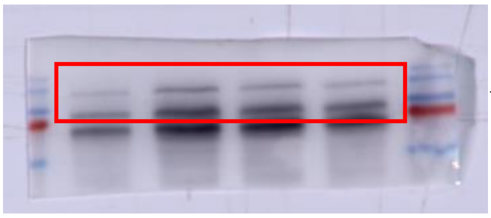

N-cadherin (100 kDa)

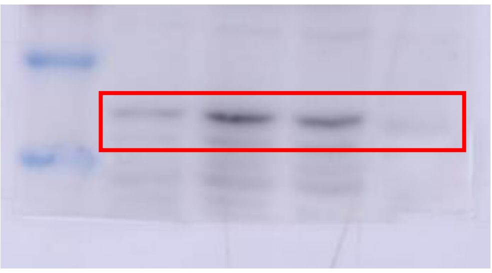

Vimentin (54 kDa)

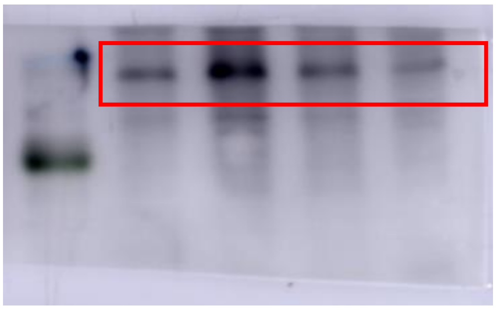

Snail (29 kDa)

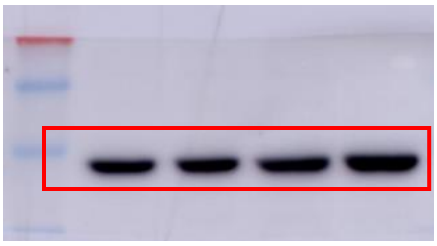

β-actin (42 kDa)

Full unedited gel for Figure 4C

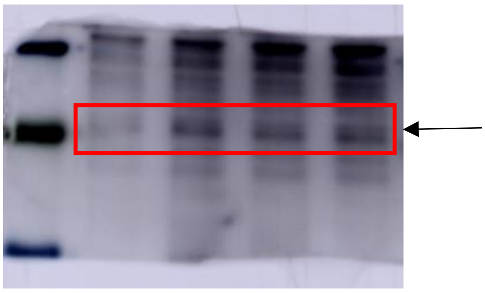

FGFBP1 (26 kDa)

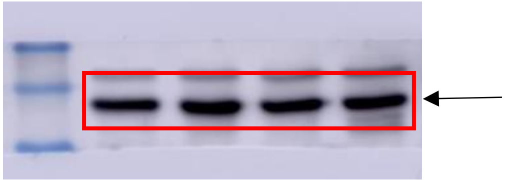

β-actin (42 kDa)

Full unedited gel for Figure 5A

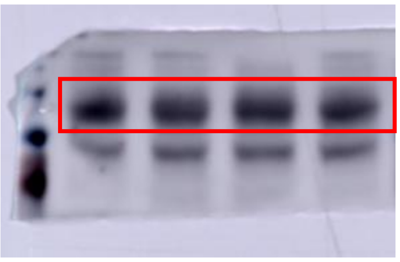

FGFR1 (120 kDa), Left

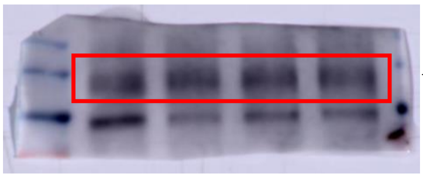

FGFR1 (120 kDa), Right

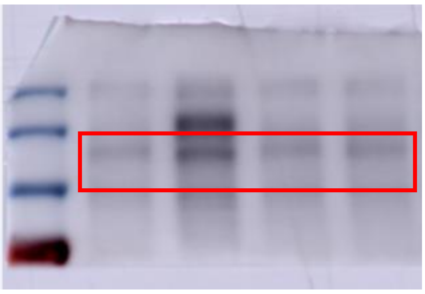

p-FGFR1^{Y654} (120 kDa), Left

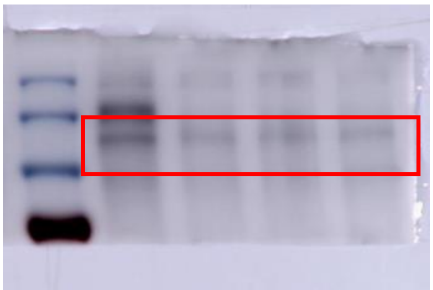

p-FGFR1^{Y654} (120 kDa), Right

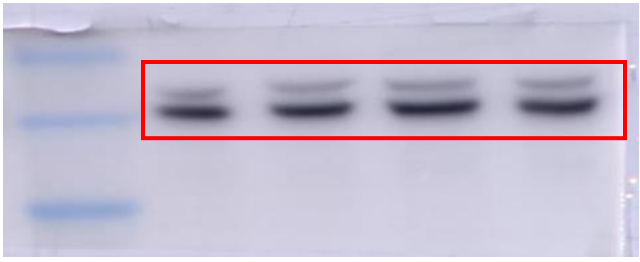

Erk1/2 (44，42 kDa), Left

Full unedited gel for Figure 5A

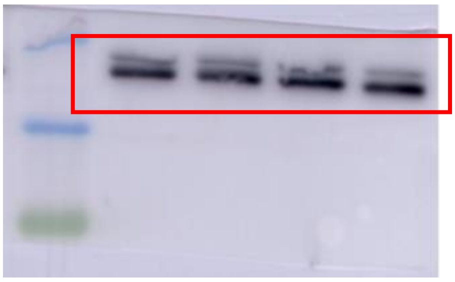

Erk1/2 (44，42 kDa), Right

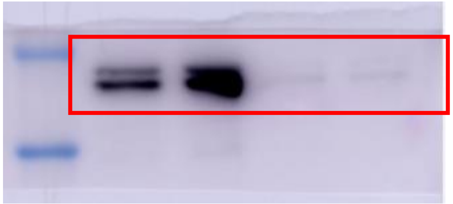

p-Erk1/2$^{Thr202/Tyr204}$ (44， 42 kDa), Left

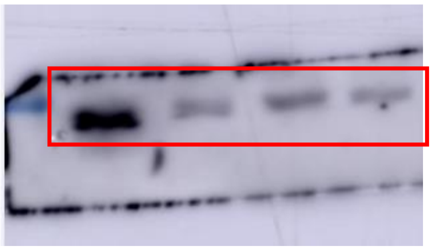

p-Erk1/2$^{Thr202/Tyr204}$ (44， 42 kDa), Right

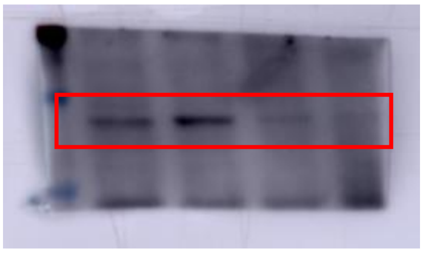

EGR1 (58 kDa), Left

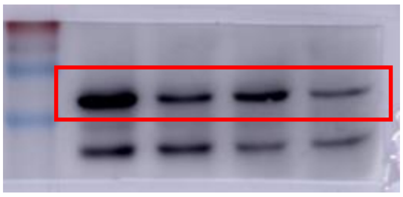

EGR1 (58 kDa), Right

Full unedited gel for Figure 5A

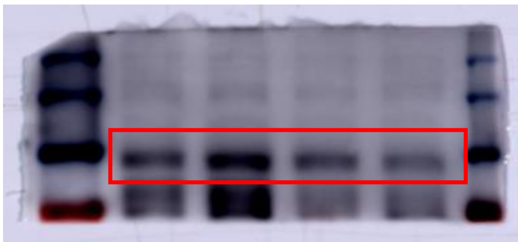

FAPα (90 kDa), Left

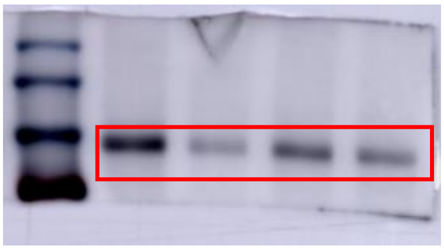

FAPα (90 kDa), Right

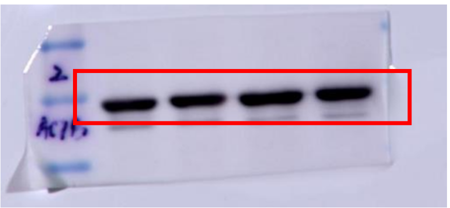

β-actin (42 kDa), Left

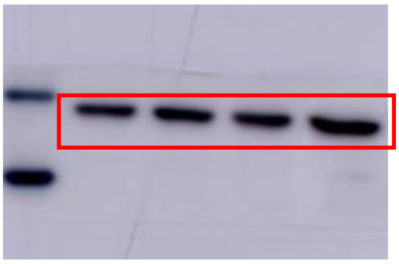

β-actin (42 kDa), Right

Full unedited gel for Figure 5B

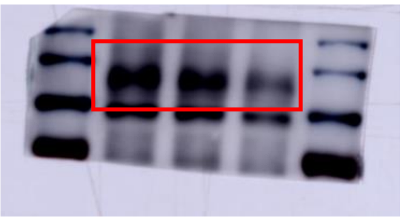

FGFR1 (120 kDa), Left

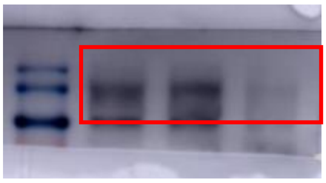

FGFR1 (120 kDa), Right

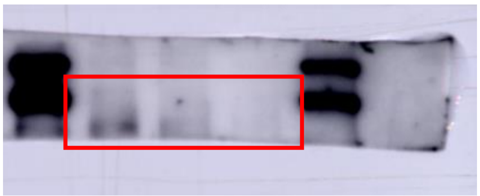

p-FGFR1^{Y654} (120 kDa), Left

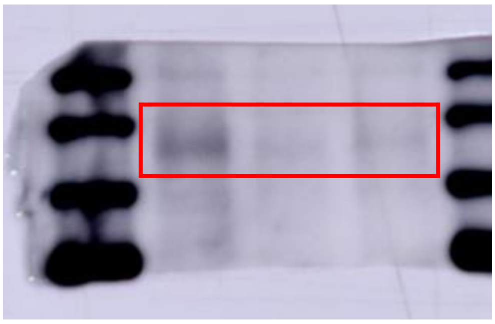

p-FGFR1^{Y654} (120 kDa), Right

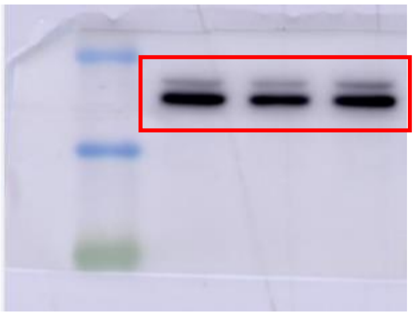

Erk1/2 (44， 42 kDa), Left

Full unedited gel for Figure 5B

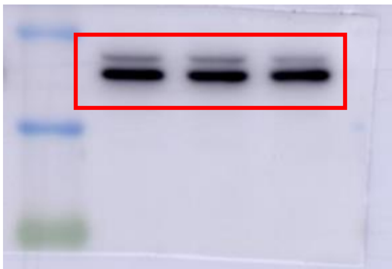

Erk1/2 (44，42 kDa), Right

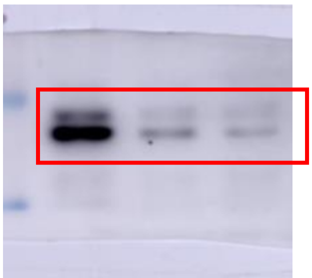

p-Erk1/2$^{Thr202/Tyr204}$ (44， 42 kDa), Left

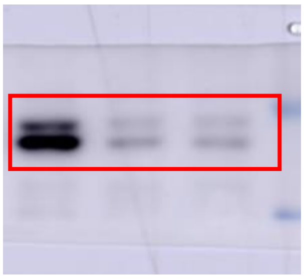

p-Erk1/2$^{Thr202/Tyr204}$ (44， 42 kDa), Right

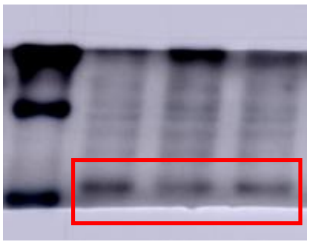

EGR1 (58 kDa), Left

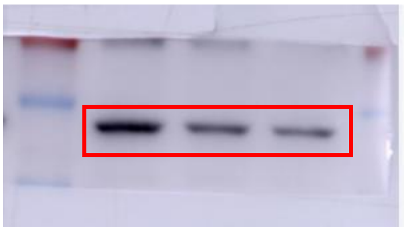

EGR1 (58 kDa), Right

Full unedited gel for Figure 5B

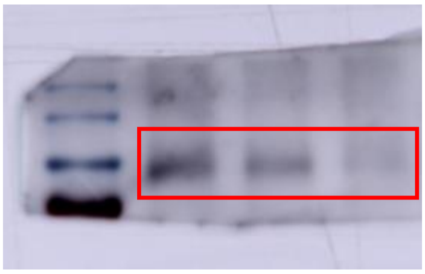

FAPα (90 kDa), Left

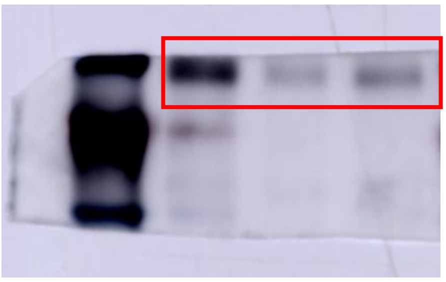

FAPα (90 kDa), Right

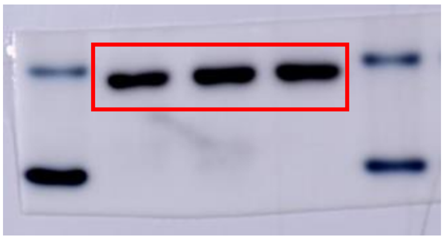

β-actin (42 kDa), Left

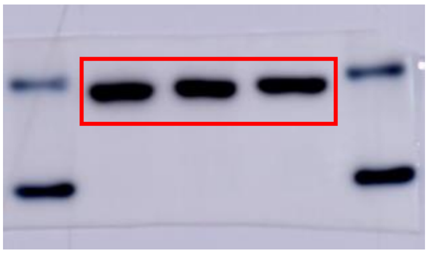

β-actin (42 kDa), Right

Full unedited gel for Figure 5C

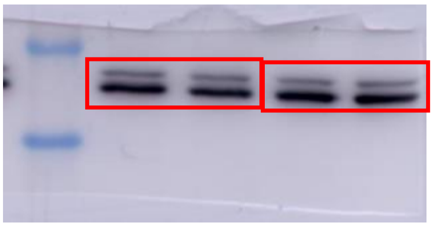

Erk1/2 (44， 42 kDa)

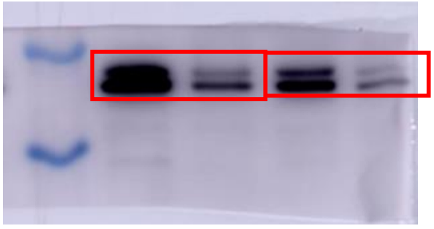

p-Erk1/2$^{Thr202/Tyr204}$ (44， 42 kDa)

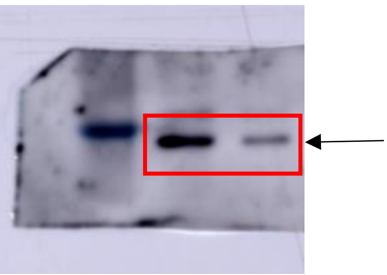

EGR1 (58 kDa), Left

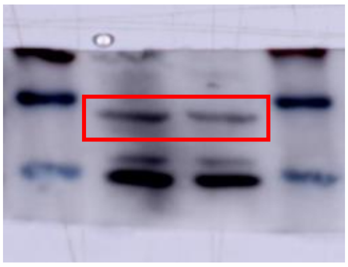

EGR1 (58 kDa), Right

Full unedited gel for Figure 5C

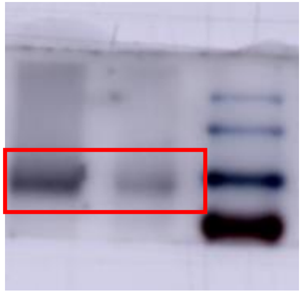

FAPα (90 kDa), Left

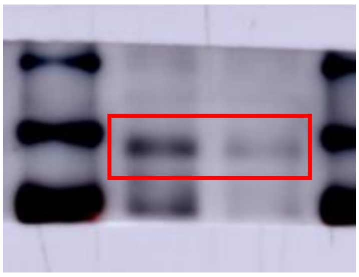

FAPα (90 kDa), Right

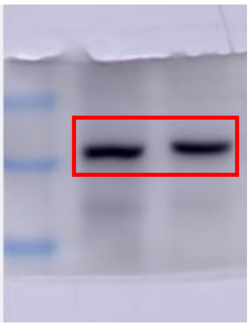

β-actin (42 kDa), Left

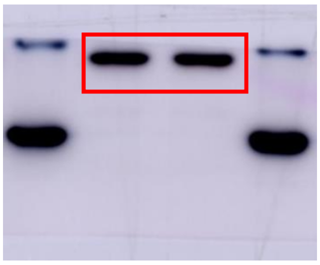

β-actin (42 kDa), Right

Full unedited gel for Figure 5D

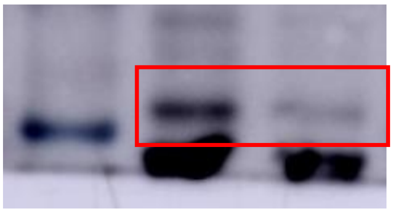

EGR1 (58 kDa), Left

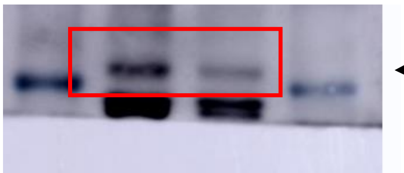

EGR1 (58 kDa), Right

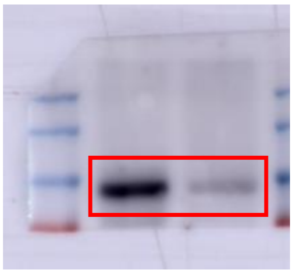

FAPα (90 kDa), Left

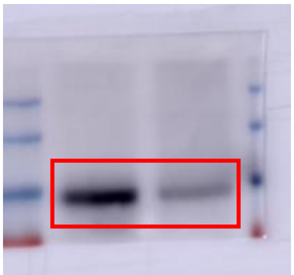

FAPα (90 kDa), Right

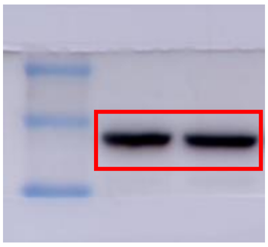

β-actin (42 kDa), Left

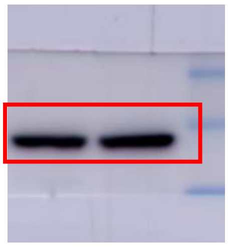

β-actin (42 kDa), Right

Full unedited gel for Figure 6J

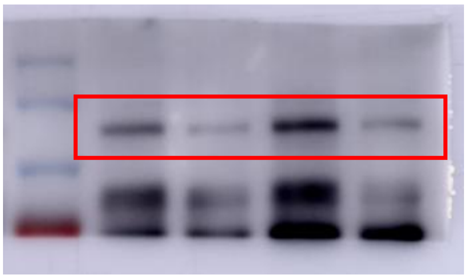

E-cadherin (97 kDa)

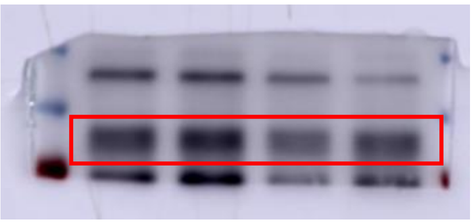

N-cadherin (100 kDa)

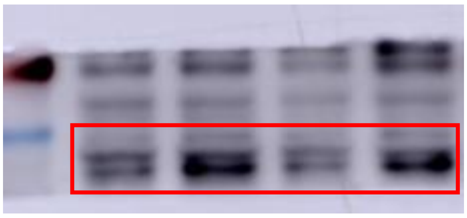

Vimentin (54 kDa)

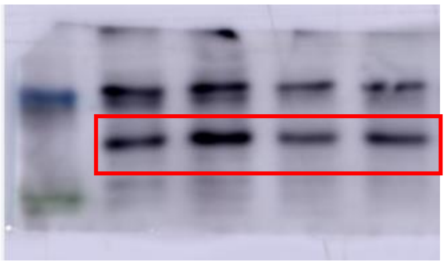

Snail (29 kDa)

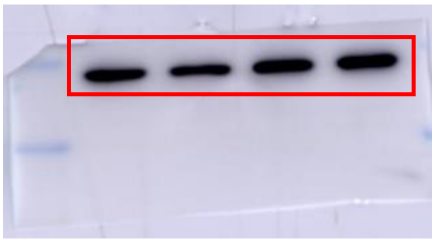

β-actin (42 kDa)

Full unedited gel for Supplemental Figure 3B

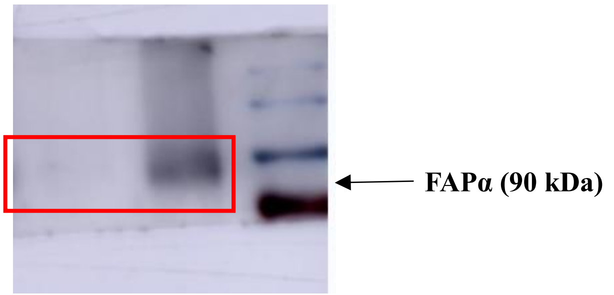

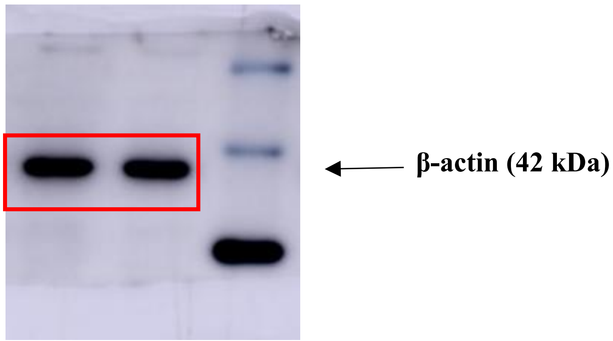

Full unedited gel for Supplemental Figure 4E

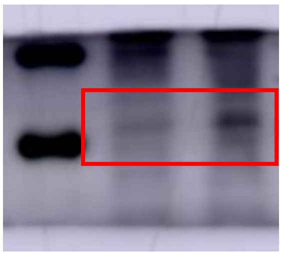

FGFBP1 (26 kDa), Left

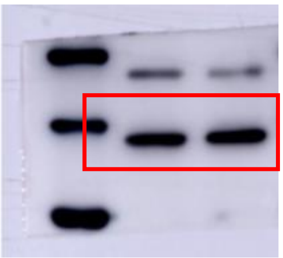

β-actin (42 kDa), Left

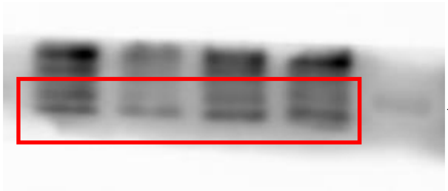

FGFBP1 (26 kDa), Right

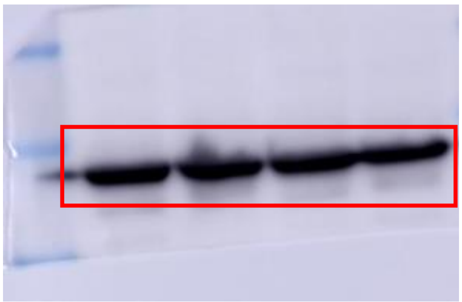

β-actin (42 kDa), Right

Full unedited gel for Supplemental Figure 7A

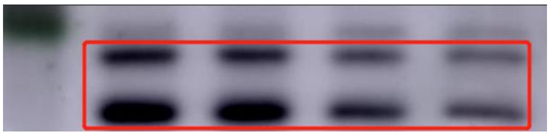

FGF2 (24,22,18 kDa)

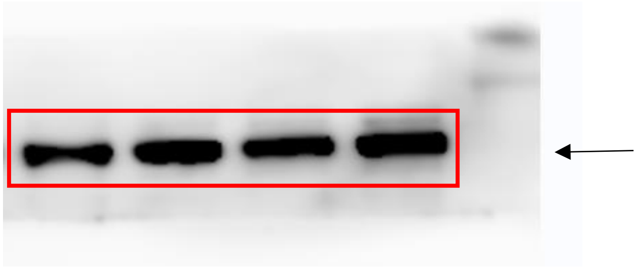

β-actin (42 kDa)

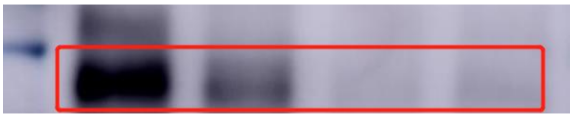

FGFR1 (145,120 kDa)

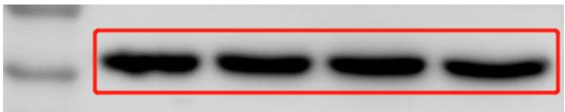

β-actin (24,22,18 kDa)

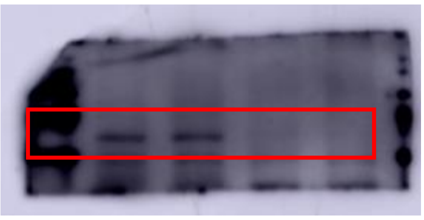

EGR1 (58 kDa)

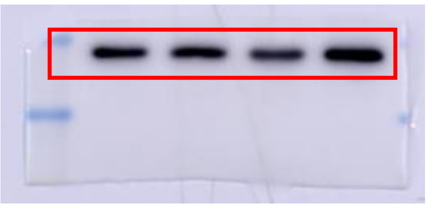

β-actin (42 kDa)

Full unedited gel for Supplemental Figure 11A

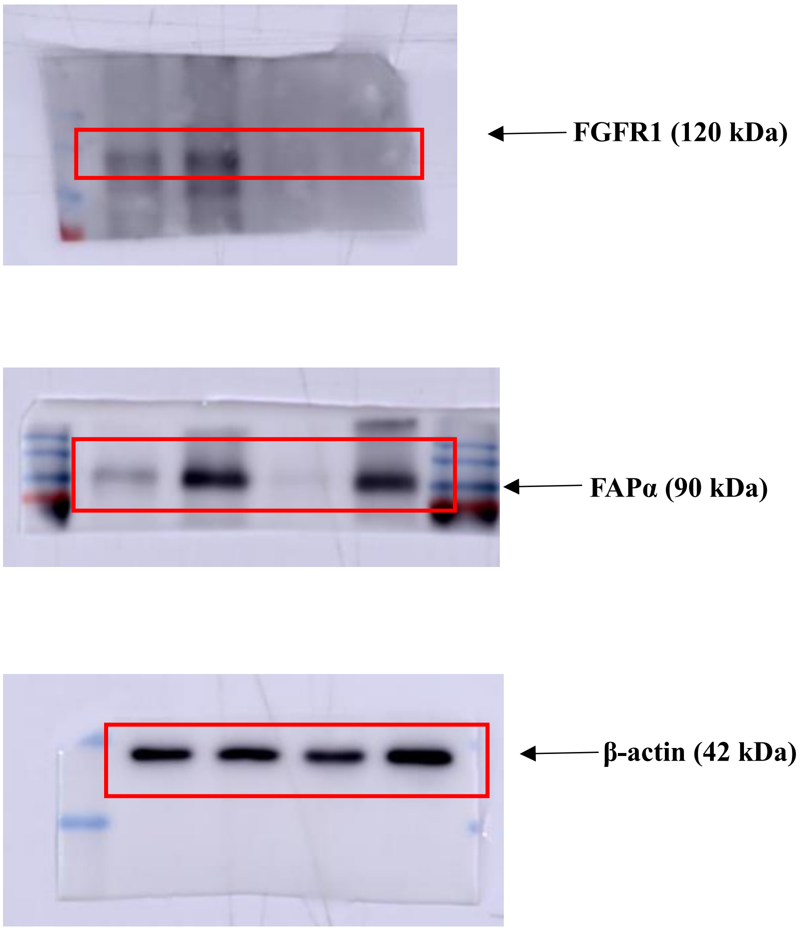

Full unedited gel for Supplemental Figure 14C

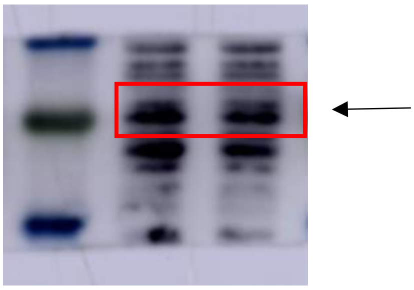

FGFBP1 (26 kDa)

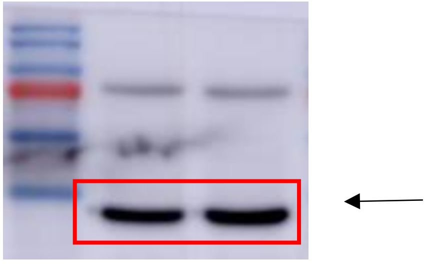

β-actin (45 kDa)

Full unedited gel for Supplemental Figure 14D

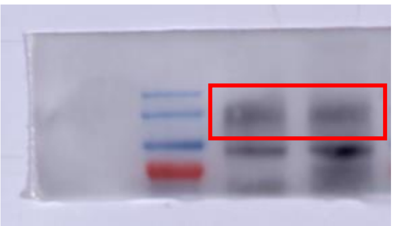

FGFR1 (120 kDa), Left

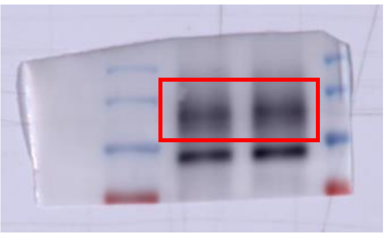

FGFR1 (120 kDa), Right

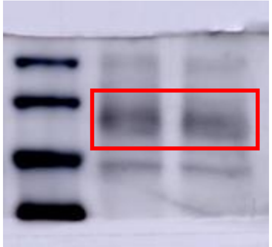

p-FGFR1^{Y654} (120 kDa), Left

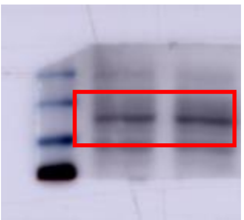

p-FGFR1^{Y654} (120 kDa), Right

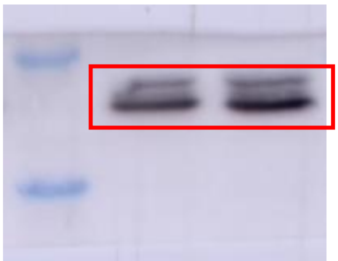

Erk1/2 (44， 42 kDa), Left

Full unedited gel for Supplemental Figure 14D

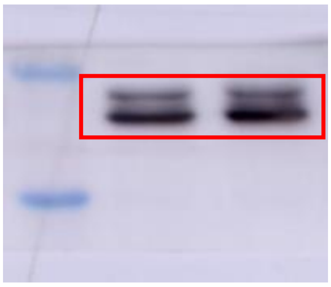

Erk1/2 (44，42 kDa), Right

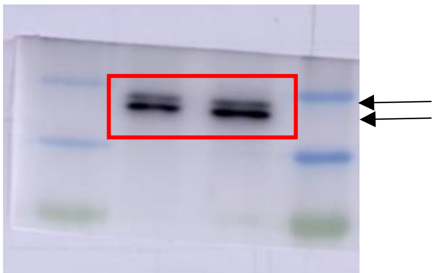

p-Erk1/2$^{Thr202/Tyr204}$ (44， 42 kDa), Left

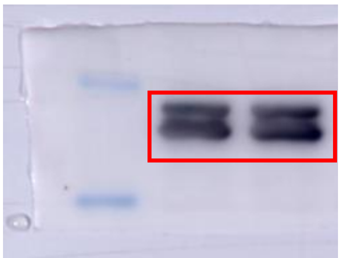

p-Erk1/2$^{Thr202/Tyr204}$ (44， 42 kDa), Right

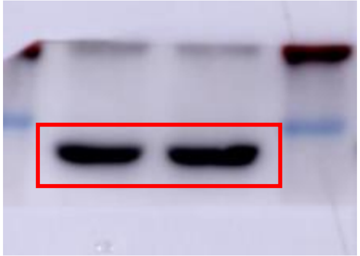

EGR1 (58 kDa), Left

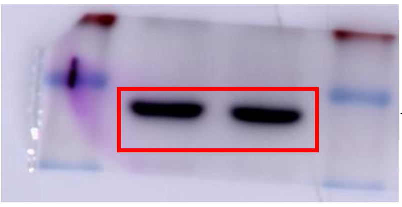

EGR1 (58 kDa), Right

Full unedited gel for Supplemental Figure 14D

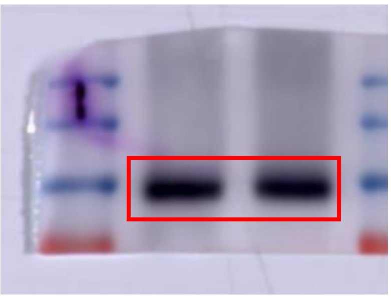

FAPα (90 kDa), Left

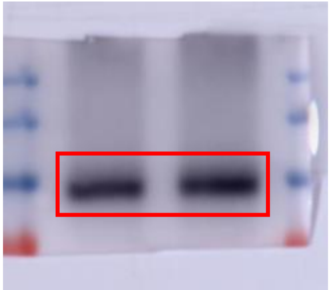

FAPα (90 kDa), Right

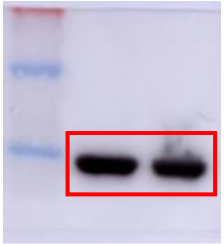

β-actin (42 kDa), Left

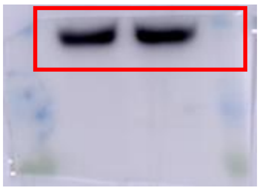

β-actin (42 kDa), Right

Full unedited gel for Supplemental Figure 15B

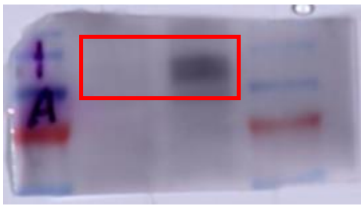

HIF-1α (120 kDa)

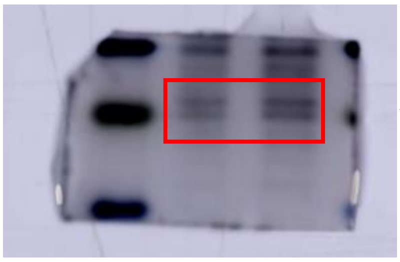

FGFBP1 (26 kDa)

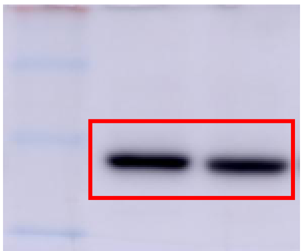

β-actin (42 kDa)

Full unedited gel for Supplemental Figure 15C

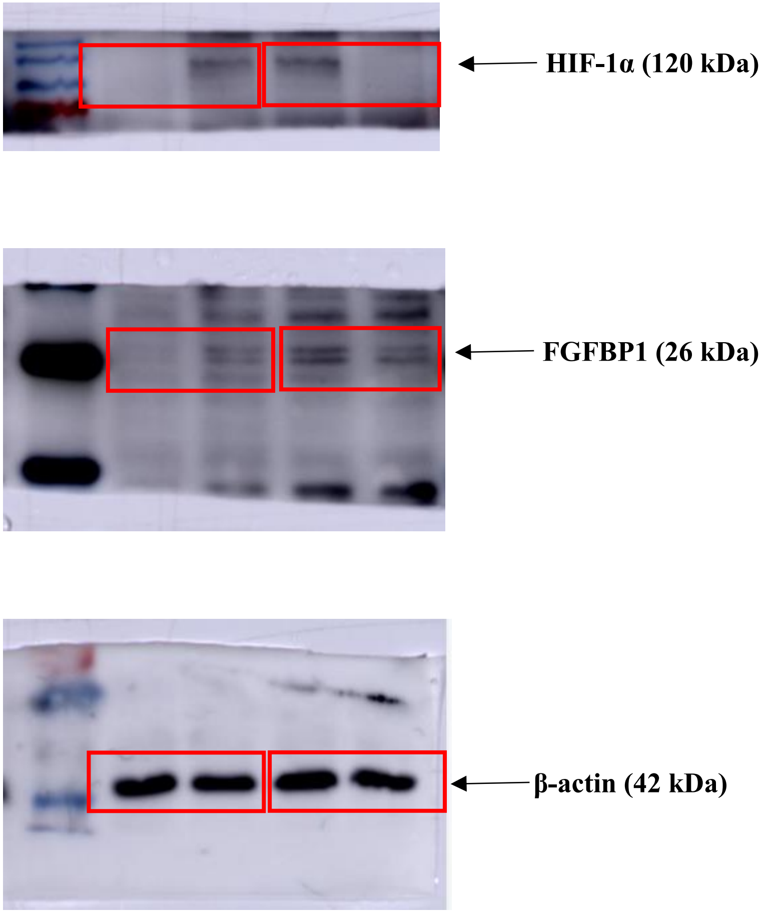

Supplement: Supplemental data [file jci-132-157399-s067.pdf]
